# Inhalable antibiotic resistomes emitted from hospitals: metagenomic insights into bacterial hosts, clinical relevance, and environmental risks

**DOI:** 10.1186/s40168-021-01197-5

**Published:** 2022-01-27

**Authors:** Dong Wu, Ling Jin, Jiawen Xie, Hang Liu, Jue Zhao, Dan Ye, Xiang-dong Li

**Affiliations:** 1 Department of Civil and Environmental Engineering, The Hong Kong Polytechnic University, Hung Hom, Kowloon, Hong Kong, China; 2 Shanghai Engineering Research Center of Biotransformation of Organic Solid Waste, School of Ecological and Environmental Science, East China Normal University, Shanghai, 200241 China; 3 Department of Health Technology and Informatics, The Hong Kong Polytechnic University, Kowloon, Hong Kong, China; 4 University Research Facility in Chemical and Environmental Analysis, The Hong Kong Polytechnic University, Kowloon, Hong Kong, China; 5 The First Affiliated Hospital of Guangzhou Medical University, 151 West Yanjiang Road, Guangzhou, 440104 China

**Keywords:** Antibiotic resistome, Hospital PM_2.5_, ARG-hosting bacteria, Healthcare-associated infection, AMR risk

## Abstract

**Background:**

Threats of antimicrobial resistance (AMR) to human health are on the rise worldwide. Airborne fine particulate matter (PM_2.5_), especially those emitted from hospitals, could serve as a substantial yet lesser-known environmental medium of inhalable antibiotic resistomes. A genome-centric understanding of the hosting bacterial taxa, mobility potential, and consequent risks of the resistomes is needed to reveal the health relevance of PM_2.5_-associated AMR from clinical settings.

**Results:**

Compared to urban ambient air PM_2.5_, the hospital samples harbored nearly twice the abundance of antibiotic resistantance genes (ARGs, ~ 0.2 log_10_(ARGs/16S rRNA gene)) in the summer and winter sampled. The profiled resistome was closely correlated with the human-source-influenced (~ 30% of the contribution) bacterial community (Procrustes test, *P* < 0.001), reflecting the potential antibiotic-resistant bacteria (PARB), such as the human commensals *Staphylococcus* spp. and *Corynebacterium* spp. Despite the reduced abundance and diversity of the assembled metagenomes from summer to winter, the high horizontal transfer potential of ARGs, such as the clinically relevant *bla*_OXA_ and *bac*A, in the human virulent PARB remained unaffected in the hospital air PM samples. The occurring patterns of β-lactam resistance genes and their hosting genomes in the studied hospital-emitting PM_2.5_ were closely related to the in-ward β-lactam-resistant infections (SEM, std = 0.62, *P* < 0.01). Featured with more abundant potentially virulent PARB (2.89 genome copies/m^3^-air), the hospital samples had significantly higher resistome risk index scores than the urban ambient air samples, indicating that daily human exposure to virulent PARB via the inhalation of PM_2.5_ was ten times greater than from the ingestion of drinking water.

**Conclusions:**

The significance of AMR in the studied hospital-emitting PM_2.5_ was highlighted by the greater abundance of ARGs, the prevalence of potentially virulent PARB, and the close association with hospital in-ward β-lactam infections. A larger-scale multi-source comparison of genome-resolved antibiotic resistomes is needed to provide a more holistic understanding to evaluate the importance of airborne AMR from the “One-Health” perspective.

Video Abstract

**Supplementary Information:**

The online version contains supplementary material available at 10.1186/s40168-021-01197-5.

## Background

The accelerating propagation of antimicrobial resistance (AMR) is a threat to global public health [1]. AMR annually causes 700,000 deaths worldwide, and the death toll may exceed 10 million by the middle of the twenty-first century if current practices on the use of antibiotics remain unchanged [2, 3]. A quintessential “One Health” issue, AMR can be developed, transmitted, and prevail in the environment via multiple pathways, thereby constituting an integral dimension of the human-animal-environment loop [4, 5]. In comparison with soil, water, and waste [6], AMR materials in ambient air are more pervasively and closely interconnected with human beings [7]. In particular, AMR associated with airborne fine particulate matter (PM_2.5_) exacerbates this health issue because PM_2.5_ can penetrate deeply into the alveolar region and even enter the bloodstream [8, 9]. Inhaled antibiotic resistantance genes (ARGs) have been found to expose human beings to a concentration of 10^2–3^ copies/m^3^-air [10], and their pathogenic bacteria hosts could increase the chances of resistant infections through air inhalation. The enduring existence of inhalable ARGs, together with human pathogens like *Streptococcus pneumonia* (0.05%) and *Aspergillus fumigatus* (5.8% of the whole microbiome), has been detected in severe PM_2.5_ pollution days [11]. These airborne particles accommodate dynamic compositions of microbes originating from a variety of emission sources (e.g., human feces and husbandry waste) due to mechanic agitation, wastewater aeration, and biosolid aerosolization [12]. The pathogens therein included *Pseudomonas aeruginosa*, *Stenotrophomonas maltophilia*, and *Talaromyces marneffeiin*. Inhalation of these pathogens as part of airborne particles may lead to an increased risk of respiratory infections [12–14].

The profiles of airborne ARGs are generally impacted by the features of their emission sources and atmospheric conditions [14]. Compared to other heavily AMR-laden environments (e.g., feedlots, sewage treatment works, landfill sites) [15–17], clinical settings are generally characterized by the more intensive use of frontline antibiotics and frequent occurrence of human bacterial pathogens [18, 19]. These issues are particularly relevant to large urban hospitals in fast-developing countries, where the overuse of antibiotics is commonplace [20], and healthcare-associated infections (HAIs) and patient overcrowding conditions are reportedly severe [21]. In one study focusing on the clinical settings [22], bioaerosol concentrations were found to be higher in the inpatient areas (115 ± 13 cfu/m^3^) than in other sites (80 ± 7 cfu/m^3^), and some human opportunistic pathogenic commensals (e.g., *Streptococcus* spp. and *Staphylococcus* spp.) were observed in the bioaerosols of inpatient wards and other public areas of hospitals. These airborne bacteria associated with inhalable particles (e.g., PM_2.5_ and PM_10_) that are (potentially) virulent to humans can be transmitted from the air to the human respiratory system via inhalation [23–26]. Moreover, mobile genetic elements (MGEs) in the air can facilitate the dissemination of ARGs to airborne bacteria via horizontal gene transfer (HGT) [7]. Hence, the co-existence of airborne ARGs, MGEs [27–29], and bioaerosols [22] from clinical sources may facilitate the movement of potential antibiotic-resistant bacteria (PARB) in the airborne particles emitted from hospitals. This situation should be systemically studied.

PM_2.5_-associated ARGs have been detected at comparatively higher levels in air samples close to hospitals than to other urban areas (0.4 *vs*. 0.1 ARGs/16s rRNA gene in relative abundance) [27], implying that hospital-emitted air particles may be the main contributors to ARGs in the bioaerosol matrix. However, little is known about (i) the genome-centric profile of resistomes, HGT potentials, and associated virulence; (ii) linkages with hospital ward AMR infection cases; and (iii) AMR risk levels relative to other environmental settings and exposure pathways. Moreover, to date, metagenomic sequencing and relevant analyses on air samples, especially on ambient airborne PM_2.5_ [30], have yet to be systematically conducted. As such, to address these key scientific issues, PM_2.5_ samples emitted from a large urban hospital were collected. In the meantime, the datasets concerning HAI cases and antibiotic consumption from the hospital’s administration department were retrieved for this study. The most accessible metagenomic sequencing datasets were also obtained from urban ambient airborne PM_2.5_ and drinking water, which were compiled with the hospital samples to characterize the resistome and hosting bacterial taxa and to explore the AMR health risks across different environments and exposure pathways.

## Methods

### PM_2.5_ sample collection and pretreatment

A high-volume PM_2.5_ sampler (ASM-1, Mingye Inc. China) was set upon the ventilation outfalls on the rooftop of the inpatient building of a large urban hospital in Guangzhou, China (Supplementary Information; Additional file 1: Fig. S1). The sampling ventilation outfalls are connected with the vent pipes linking to the inpatient zones and emergency rooms of Department of Pulmonology and Critical Care Medicine. The emitted bioaerosols from these places are expected to contain typical human-associated airborne microbiomes and can better represent the air-transmission health risks with clinical relevance. On the one hand, it may reflect what is in the inpatient zone with nosocomial airborne transmission. On the other hand, it may also link to what the neighboring community could be exposed to as a potential AMR source. This hospital well known for its pulmonology medicine serves more than 150,000 inpatients annually, which is typical for a large municipal public hospital in China. Ambient air was drawn at an average flow rate of 1 m^3^/min for 24 h (10:00 AM to 10:00 AM) per sampling day, corresponding to approximately ~ 1500 m^3^ of air flow-through per sampling day. The PM_2.5_ sampling campaign was conducted in two separate periods in the year 2019: June to August (summer, *n* = 10) and late September to December (winter, *n* = 9). Two to three samples were collected on a weekly basis in each sampling month. In addition, ambient air PM_2.5_ samples collected from Guangzhou city (Tianhe (TH) and Conghua (CH) Districts) from April 2016 to May 2017 (same sampling procedure to the hospital samples, *n* = 10) were used for an urban comparison study in the present research. The sampling date of all samples was provided in Additional file1 (Table S1). All the filters were sterilized by baking in a Muffle furnace at 500 °C for 5 h prior to sampling. Each sterilized filter was packaged in sterilized aluminum foil and stored in a humidity-controlled chamber until being loaded into the filter cartridge.

The collected PM_2.5_ filters were packaged in the sterilized aluminum foil and zip bags and were immediately transferred to the lab in an ice box. The pretreatment of filter samples followed the previously published protocol with modifications [31]. Half of the A4-size filter was sonicated with sterilized phosphate-buffered saline (PBS). The particulates that were deposited in PBS-extract aliquots were filtered through a PES membrane disc-filter (0.2 μm × 47 mm, Supor 200, PALL Co., USA). The disc-filters that were obtained were stored at − 20 °C prior to the extraction of DNA. Details of the sample collection and pretreatment procedures of all PM_2.5_ samples are provided in the Supplementary Information (SI-Additional file 1: SI-1).

### DNA extraction and meta-sequencing

A FastDNA Spin Kit (MP, USA) was used to extract metagenomic DNA from the obtained disc-filters according to the manufacturer’s instructions, with modified binding and purification steps where the DNA binding matrix was replaced by an Agencourt AMPure XP bead to improve the yield of DNA (Beckman Coulter, USA). The extracted DNA samples were subjected to paired-end sequencing (150 bp) on an Illumina Hiseq X Ten platform. The DNA samples were checked by using agarose gel electrophoresis and a Qubit3.0 Fluorometer (Thermo Fisher Scientific, USA). Samples showing limited degradation and a sufficient amount of dsDNA were used for library construction and metagenomic sequencing. Details of the DNA extraction/yields are provided in the SI-Additional file 1 (Table S1). The clean data have been uploaded to the NCBI with the accession numbers PRJNA726763 (hospital-specific air PM_2.5_) and PRJNA719719 (urban ambient air PM_2.5_).

### Identification of ARGs and taxonomic affiliation

Raw reads from each sample were initially processed using FASTP to remove low-quality sequences (≥ Q15) [32]. The deepARG short-read module (v0.18) pipelines were applied to map the profile of the antibiotic resistomes (identity = 80%, probability = 0.8, E-value = 1e−10), and the abundance of identified ARGs was normalized to the sequence number of the 16S rRNA gene (16s identity threshold 85%, Supplementary Information; SI-Additional file 1: SI-2 and Additional file 2). MetaPhlan3 (v3.0.6) was used to obtain the taxonomic information (from kingdom to species) of the samples by selecting --bowtie2out (taxa marker gene database: mpa_v30_CHOCOPhlAn_201901_marker_info.txt.bz2) output files with default parameters (SI-Additional file 3) [33].

### Assembly of metagenomic bins and bacteria source tracking

The filtered clean reads were grouped by sampling periods/seasons (SI-Additional file 1: Table S1) and were co-assembled using MEGAHIT v1.13 with default parameters [34]. These co-assembled contigs were clustered to recover metagenomes using MaxBin, metaBAT, and CONCOCT [35–37] by using MetaWRAP (v1.3.2, contig length ≥ 1000 bp) [38]. The assembled bacterial genomes were further refined to produce high-quality individual genomes using the built-in refining module of MetaWRAP, with the selection criteria of > 50% completeness and < 5% contamination. Following that procedure, all metagenome data were refined to remove redundant assemblages and then were annotated for taxonomic classifications by using the Genome Taxonomy Database (GTDB; v1.4.0) [39]. To analyze the potential sources of the bacteria loaded on the air PM_2.5_ samples, 16S rRNA sequences were extracted from metagenomic reads by using SortMeRNA (version 2.1b) [26]. The silva-bac-16s-id90.fasta and Greengenes 13.8 99_otus.fasta databases were used with –fastx –paired_in -blast 1 -num_alignments 1 parameter settings. Extracted 16S rRNA sequences were then analyzed by QIIME2-vesearch (v2020.11) using a closed reference against Greengenes 13.8 to generate operational taxonomic unit (OTU) tables for all of the tested samples (identity cutoff = 0.97). These tables were merged with a table of 16S rRNA gene amplicon studies downloaded from the Earth Microbiome Project (ftp://ftp.microbio.me/emp/release1/otu_tables/closed_ref_greengenes/emp_cr_gg_13_8.subset_5k.biom). This table was further filtered to remove OTUs with frequencies of less than two. The bacteria attributable to different environmental biomes at the genus level were estimated using SourceTracker2 with default parameters [40].

### Intragenomic co-existence with VFs and MGEs

The co-assembled scaffolds of all metagenomic genomes were initially processed by Prodigal (v2.6.3; -c -p meta mode) to predict open reading frames (ORFs) [41]. With the application of CD-HIT (v4.6) [42], these ORF sequences were further clustered under the criteria of 90% identity over the ORFs with > 90% coverage in the length of the sequences (> 250 bp). The clustered ORFs were aligned with existing ARGs (v1.1.1.A.fasta; https://bench.cs.vt.edu/ftp/argminer/release/) and MGE databases (https://bench.cs.vt.edu/ftp/data/databases/) via DIAMOND (v2.0.9) by using the parameters of alignment = 1, threshold value = 1e−10, identity > 70%, and query coverage > 50%. Metagenomic-assembled genomes with queried scaffolds that carried no less than one ARG-like gene were identified as potential antibiotic-resistant bacteria (PARB) [43]. To screen the human virulence factor (HVF) genes, ABRicate pipelines (v0.9.9) were utilized (default parameters) with reference to its built-in HVF database (VFDB_setB_nt.fas; http://www.mgc.ac.cn/VFs/download.htm; database update: 2019-Jul-28). The identified PARB containing no less than one human virulent factor (HVF) gene was regarded as the HVF-PARB.

### Quantification of metagenomes and hosted ARGs

The Quant_bin module of MetaWRAP (v1.3.2) was used to calculate the abundance of constructed genomes with default parameters [38]. Generally, read counts for the assembly of each sample were generated (with clean reads) using Salmon (v0.13.1) [44], which provided a relative abundance table (genome copies per million reads (ppm)) for each scaffold across different samples. For each genomic bin, the abundance of the containing contigs was summed and normalized to the total mapping read numbers in different samples (genome copies/ppm reads). To quantify the identified ARG sequences located in each binning genome, Seqkit (v0.16.0), which was based on the DIAMOND output (query sequence information) files, was used in the study. The compiled sequences of the hospital and urban files were mapped to the extracted target genes using Bowtie2 (v2.3.5) with default parameters [45]. The generated SAM files were further processed using the built-in pipelines (pileup.sh) of BBmap (v38.87). The calculated average coverage folds were normalized to their sequencing size, and then were used to represent the abundance of target genes [46]. The details and equations are provided in the Supplementary Information (Eq S1, SI-Additional file 1).

### Retrieval of clinical and sequencing data and evaluation of potential health risks

Clinical data on the HAI cases and consumption of antibiotic drugs during the sampling period were extracted from the monthly published reports, which were issued by the administrative department of the sampled hospital. Metagenomic sequencing data related to drinking water (PRJNA305188) were retrieved from the National Center for Biotechnology Information (NCBI). The data on the ingestion AMR hazards and risks arising from the drinking water were then compared with the data from the airborne (hospital and urban) PM_2.5_ samples. MetaCompare pipelines (git cloned from https://github.com/minoh0201/MetaCompare), assessing the abundance and mobility of antibiotic resistomes and their hosts’ pathogenicity, were used to calculate the AMR risk (a relative-risk index generated) of the resistome with default parameters [47]. To quantify the bacteria into the volume unit (e.g., m^3^), metagenome-based analyses were conducted. For a target environmental compartment, the relative abundance of each genome was averaged on all samples (genome copies/ppm). The mean abundance was multiplied with the sequencing read numbers used in the bin-assemblage of each sample (ppm/sample), and the resulting figure was further normalized to the volume (i.e., m^3^ air/sample, L water/sample) of samples, which resulted in the concentrations of the target bacteria metagenomes (genome copies/ m^3^ air or genome copies/L water). Details on the processing of the data and the used equations are provided in the Supplementary Information (Additional file 1: SI-3).

### Statistics

Descriptive statistics for all data were generated using Excel 2010 (Microsoft Corp., USA). Mean values and standard deviations were rounded to two decimal places. In each dataset, the outliers were detected by using the built-in dataset description function (± 1.5 × interquartile (25–75%) ranges; Tukey’s Hinges) of SPSS Statistics 22 (IBM, USA) and were excluded for the further statistical analyses. The advanced statistical analyses were conducted using R3.5.2 (https://www.r-project.org/). Details on specific methods and usages are provided in the Supplementary Information (Additional file 1: SI-3). Statistical significance was always defined by 95% confidence intervals (*P* < 0.05).

## Results and discussion

### Broad-spectrum profile of the ambient air PM_2.5_ resistome

**Fig. 1:**
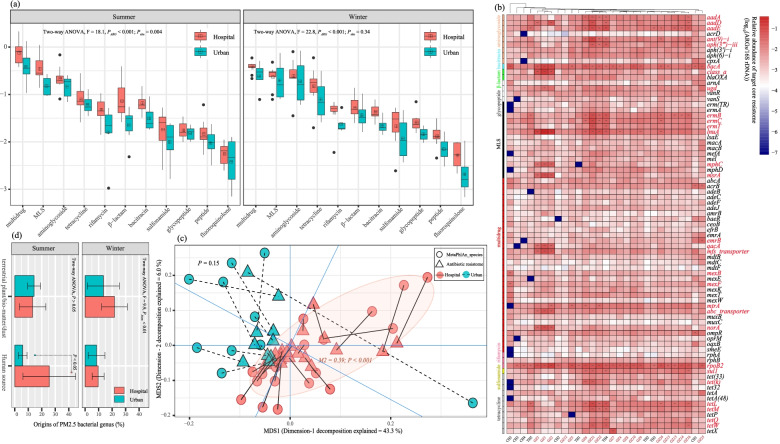
**a** Relative abundance of airborne ARGs in hospital and urban ambient air PM_2.5_ samples in summer and winter. Eleven resistance types of ARGs were calculated as the major components of the resistome (> 99% of the total abundance). **b** The core resistome of PM_2.5_ contained 88 subtypes of ARGs, which occurred in more than 90% of the samples. The ARGs with relative abundance > − 2.0 log_10_(ARGs/16S rRNA gene) are annotated in red. **c** Bacterial taxa (species) classified using MetaPhlan3 were significantly correlated with the hospital PM_2.5_ resistome. **d** Percentage of bacterial genera attributable to two predominant environmental setting origins (%), namely terrestrial bio-matter/plants and human-associated waste (feces, sebum, saliva), as determined using SourceTracker2

Eleven dominant types of ARGs (> 99% of the resistome) constituted the whole resistome in the hospital and urban ambient air PM_2.5_ samples in Guangzhou city (Fig. 1a). The hospital samples harbored a significantly more abundant resistome (0.19 ± 0.14 log_10_(ARGs/16S rRNA gene)) than urban ones in total (− 0.03 ± 0.10 log_10_(ARGs/16S rRNA gene)); SI-Additional file 1: Fig. S2), regardless of season (two-way ANOVA, *P*_site_ = 0.05, *P*_season_ = 0.25). Among the specific resistance types of ARGs (Fig. 1a), multidrug resistance was the most abundant in the hospital (− 0.28 ± 0.19 log_10_(ARGs/16S rRNA gene)) and urban (− 0.50 ± 0.24 log_10_(ARGs/16S rRNA gene)) samples (two-way ANOVA, *P*_ARG_ < 0.001). As shown in Fig. 1a, ARGs encoding resistance to aminoglycoside, macrolide–lincosamide–streptogramin (MLS), tetracycline, and β-lactam were identified as major components of the resistome, generally ranging from − 0.5 to − 1.5 log_10_(ARGs/16S rRNA gene) in both the hospital and urban samples during two sampling seasons. Meanwhile, bacitracin-, rifamycin-, sulfonamide-, (glyco)peptide-, and fluoroquinolone-resistant genes were regarded as minor components in the hospital PM_2.5_ (< − 1.5 log_10_ (ARGs/16S rRNA gene)). In comparison with urban ambient air PM_2.5_ (Fig. 1a), the abundance of each AMR type was generally at a 0.2 to 0.5 order of magnitude higher, with the difference with regard to the major ARGs becoming more pronounced during the summertime, such as with the β-lactam and MLS ARGs (*T*-test, *P* < 0.05). The results are consistent with those from previous investigations of hospital-specific airborne ARGs and indicate that hospitals could be major airborne ARG hotspots in cities [27].

Moreover, a total of 88 subtypes of ARGs were consistently detected in all of the hospital and urban PM_2.5_ samples. This core resistome profile was clustered distinctively by sampling site (PERMANOVA, *F* = 3.36, *P* = 0.001, Bray-Curtis distance). The predominant ARGs of each resistance type are shown in Fig. 1b, annotated in red (> − 1.5 log_10_(ARGs/16S rRNA gene) in at least one sample). The multidrug ARGs were primarily comprised of *qacA/C* and *mtrA* (Fig. 1b), occurrences of which are usually associated with resistance to clinical disinfectants and *Mycobacterium tuberculosis* AR infections [48, 49], respectively. With regard to the rifamycin-resistant genes, which encode resistance to tuberculosis drugs, *rpoB2* were detected with a mean concentration of 1.10 ± 0.09 log_10_(ARGs/16S rRNA gene) in the hospital sample, which was significantly higher than in its urban air counterpart (one-way ANOVA, *P* < 0.001). Other clinically important ARGs, like *vanR* and u*gd* and *bla*_OXA_ and class-A resistance encoding genes with respect to (glyco)peptide and β-lactams [54], were prevalent in the hospital samples, and all of these ARGs were significantly more abundant there than in the urban air PM_2.5_ samples (one-way ANOVA, *P* < 0.05). Given their lower abundance in other ARG hotspots (e.g., domestic wastewater and feedlots) [50, 51], these ARGs may be regarded as key features in the distinct hospital-derived resistomes. In terms of sulfonamide, MLS, tetracycline, and bacitracin ARGs, the predominant components included *sul1*, *msrA*, *tetL*, and *bacA*, respectively. All of them, except for *sul1*, which is commonly detected in natural environments and encodes non-emerging AMR [52, 53], were more abundant in the hospital samples than in the urban air samples (one-way ANOVA, *P* < 0.05). This may, to a large extent, suggest an enriched resistome in air PM_2.5_ emitted from the hospital.

### Dynamic microbial community constantly associated with the antibiotic resistome in hospital PM_2.5_

The microbial communities of all of the samples were analyzed using MetaPhlan3 (Additional file 1: Fig. S3). *Actinobacteria* was the most abundant phylum in the hospital samples (32.19 ± 12.63 %), the relative abundance of which decreased slightly by 12% from summer to winter (*T*-test, *P* > 0.05). By contrast, *Firmicutes* increased significantly from ~ 22 to 40% (*P* < 0.01), mainly due to variations in its subtaxon class of *Bacilli* (Additional file 1: Fig. S4). *Proteobacteria*, primarily comprised of *Alpha*/*Gamma-Proteobacteria*, remained consistent at 15~20% throughout the whole sampling period (*P* = 0.35). By contrast, the relative abundance of the predominant bacteria, including *Actinobacteria*, *Firmicutes*, and *Proteobacteria*, fluctuated greatly in the urban air PM_2.5_ samples, ranging from 5 to 90% across all samples (Additional file 1: Fig. S3).

**Fig. 2:**
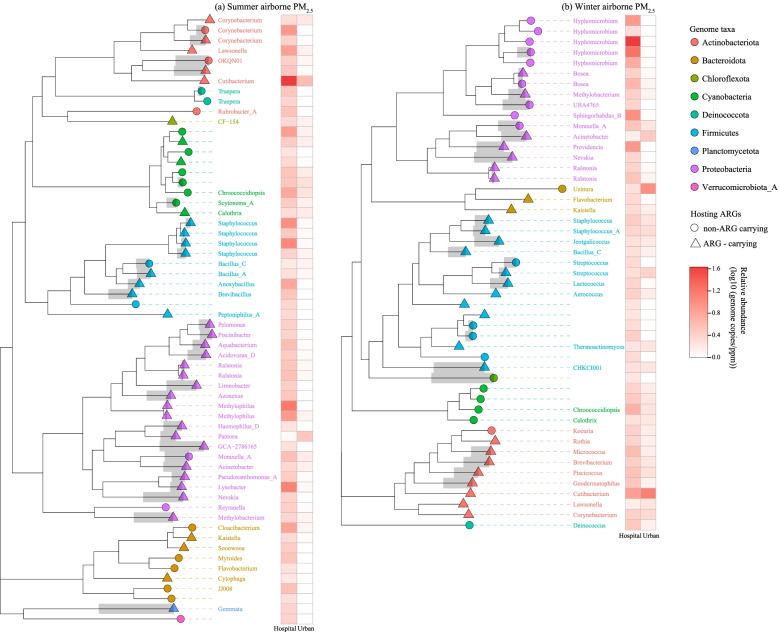
Distribution and phylogenic trees of the assembled metagenomes in summer (**a**) and winter (**b**). The ARG-hosting and non-ARG-hosting bacterial taxa are depicted in the shapes of triangles and circles, respectively. Among them, genomes that were identified as carrying human virulent factor (HVF) genes are depicted using different shades of color and the taxa belonging to the same phylum are denoted in the same color. The relative abundances of the metagenomes (genome copies/ppm reads) are shown in proportion to the darkness of the colors in the heatmaps

Along with variations in the bacterial community, Fig. 2c shows that the profile of the resistome (ARG subtypes) was significantly correlated with the composition of the bacterial community (species level) in hospital air PM_2.5_ (Procrustes test; permutations = 999, M^2^ = 0.39, *P* < 0.001), while a close association between resistome and bacterial community was not observed in the urban samples (Procrustes test; *P* = 0.15). Previous studies have pointed out the bacterial phylogeny structures the resistome, which can be strengthened by anthropogenic influences [54–56]. In our sampled airborne PM_2.5_, some human commensal bacteria that are thought to have human (opportunistic) pathogenicity with AMR relevance, such as *Enterobacteriaceae* spp., *Propionibacterium* spp., and *Micrococcus aloeverae* [57–59], varied substantially in abundance (Fig. S5), and contributed significantly to the hospital-specific differences (LefSe, log_10_(LDA score) = 0.1, *P* < 0.001) from urban samples. The urban samples featured more environmentally prevalent bacteria, such as *Ralstonia pickettii* [60], over the two sampling seasons (Fig. S5). These results suggest that human activities could have had a more pronounced influence on the compositions of the airborne bacterial community in hospital-emitted PM_2.5_ [55]. Of particular interest, by using source-tracking based on the Earth Microbiome Project (EMP) datasets [61], we found that microbiomes associated with human sources (e.g., skin, feces, sebum) contributed up to 30% of the hospital-emitted PM_2.5_ microbiomes (Fig. 1d), which was higher than the portion from terrestrial plant/bio-matter, especially in summer (*T*-test, *P* < 0.05). Note that the available data used for source-tracking involved general human microbiome and phyllosphere-related bacterial communities at a global scale (EMP database). Sequencing bacterial compositions from the hospital-specific sources would allow for a more refined assessment of their contributions.

### Genome-resolved bacterial hosts of ARGs were more abundant and virulent in hospital PM_2.5_

To further elucidate the relationship between the characteristics of PM_2.5_-borne bacteria and ARGs, a total of 109 high-quality non-redundant genomes were constructed from two sampling seasons (summer = 60 *vs.* winter = 49). As shown in Fig. 2, the relative abundance of metagenomes generally ranged from 1 to 20 genome copies per million reads (ppm) on average, and they were significantly more abundant in the hospital samples than in the urban air samples, especially during the summer (Pairwise *t*-test, *P* < 0.05). In summertime PM_2.5,_ there were 39 bacterial genomes that were classified as potential antibiotic-resistant bacteria (PARB). Among them, *Actinobacteria* (51.9 genome copies/ppm), *Firmicutes* (32.9 genome copies/ppm), and *Proteobacteria* (58.4 genome copies/ppm) accounted for > 90% of the total abundance of all PARB metagenomic bins (Fig. 2). It is noteworthy that nearly all *Proteobacteria* and *Firmicutes* PARB bins were also identified as carrying human virulent factors (HVF). The hospital-specific HVF-PARB, including *Staphylococcus* spp. and *Corynebacterium* spp., as the predominant components of *Firmicutes* and *Actinobacteria*, respectively, were almost two orders of magnitude more abundant than in the urban samples (*T*-test, *P* < 0.01, Fig. 2a). This highlights the immediate relevance of hospital PM_2.5_ as hotspots of airborne PARB in urban environment settings.

The assembled metagenomes became less diverse in winter, and the PARB decreased by one order of magnitude overall (Fig. 2b). In the meantime, the predominant attributable sources of the PM_2.5_-borne microbiome became terrestrial ones (two-way ANOVA, F = 9.9, *P*_biome_ < 0.01, Fig. 1d), seemingly implying a less correlated relationship between ARGs and anthropogenic emissions in winter. However, the identified PARB, such as *Staphylococcus* spp. (1.96 genome copies/ppm), *Corynebacterium* spp. (1.75 genome copies/ppm), and *Bacillus* spp. (1.38 genome copies/ppm), belonging to the phyla of *Firmicutes* and *Actinobacteria* (Fig. 2b), dominated the variations in the hospital PM_2.5_ bacterial communities over two sampling seasons (LefSe, *P* < 0.001; Fig. S6). By contrast, these PARB in the urban PM_2.5_ samples were either at a low abundance (< 0.1 log_10_(genome copies/ppm reads)) or rarely detected (Fig. 2b). This is in agreement with the constant correlations between the bacterial community and the resistome in PM_2.5_ emitted from the hospital, more so than in urban ambient air (Fig. 1c).

### Human virulent bacterial metagenomes harbored highly mobile ARGs of clinical importance

**Fig. 3:**
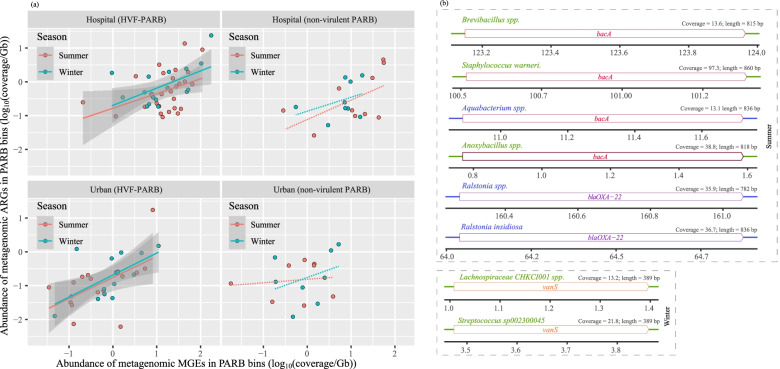
**a** The linear regression between the abundance (coverage/size) of genomic ARGs and MGEs in summer and winter. The potential antibiotic-resistant bacteria (PARB) genomes were further classified into the human virulent factors (HVF) hosting group and the non-HVF hosting group. The linear relationship fitted a significant correlation (Pearson, *P* < 0.05) and is described using a solid line with confidential intervals (gray shades), while the insignificant relationship is depicted using a dashed line. **b** ARGs associated with MGEs (on the same assembled scaffold) were shared by different bacterial genomes in winter and summer. These mobile ARGs were detected (mapped back) in the hospital samples (reads). The hosting taxa belonging to the same phylum are annotated in the same color

**Fig. 4:**
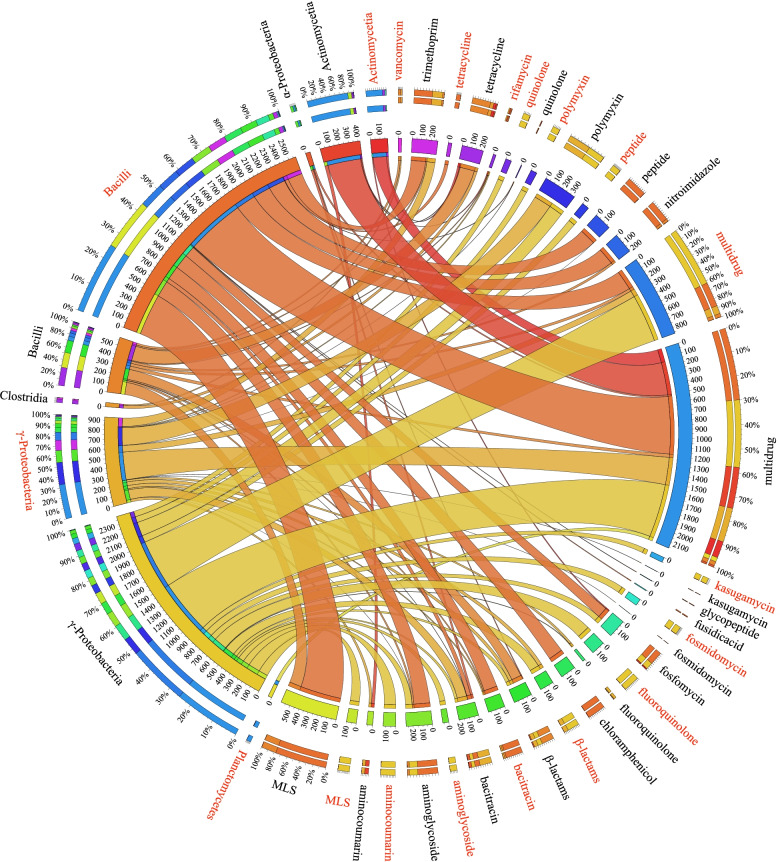
Distribution of the metagenomic antibiotic resistomes that were carried by human virulent potential antibiotic-resistant bacteria (HVF-PARB) in the hospital-specific PM_2.5._ The annotated numbers on the inner layer of the circos plot represent the abundance (coverage/size) of ARGs in the hospital samples, of which the MGE-associated ones are depicted in red. The bacterial taxa of the antibiotic resistome are annotated to the phylum level, with those labeled in red indicating those detected in summer

As shown in Fig. 3a, the identified metagenomic MGEs were significantly correlated with ARGs hosted by the HVF-PARB in both the hospital and urban air samples (Spearman, *P* < 0.001). This was irrespective of sampling season and geographic location, suggesting that HVF-PARB were the key bacteria in facilitating exchanges of ARGs [56], especially in the hospital samples (Cohen’s *D* effect size = 0.63 *vs.* 0.22, *P* < 0.001). Therefore, given the AMR importance of clinical sources and bacterial pathogenicity, ARGs carried by HVF-PARB genomes in hospital PM_2.5_ were further analyzed. Figure 4 shows that multidrug-resistant genes had the highest abundance, while the mobile genetic elements were mostly carried by γ-*Proteobacteria* and *Bacilli* in summer and winter, respectively. The macrolide–lincosamide–streptogramin (MLS)–resistant genes, as the second-most abundant metagenomic ARGs, mainly belonged to *Bacilli* in the summer samples, whereas the mobile genetic elements were carried by γ-*Proteobacteria*. Regarding the aminoglycoside-, β-lactam-, tetracycline-, and (poly)peptide- (e.g., bacitracin and polymyxin) resistant genes (Fig. 4), they were all similarly abundant in assembled HVF-PARB. Among them, β-lactam- and bacitracin-resistant genes exhibited higher mobility in *Bacilli* and γ-*Proteobacteria*. Further referring to the genetic context (Fig. 3b), the identical MGE-associated ARGs that encode resistance to broad-spectrum β-lactam and vancomycin were co-hosted by different bacterial metagenomes. Specifically, MGE-associated *bacA* and *bla*_OXA_ occurred in the human virulent *Proteobacteria* and *Firmicutes* (Fig. 3b), such as *Staphylococcus warneri*, which were commonly detected in the flora of human epithelial and mucosal membranes [62]. Those containing *bacA* were detected with a higher abundance (mapping coverage) than in other hosts in summer (Fig. 3b). In winter, the co-hosted mobile ARG became *vanS*, which was carried by HVF-*Firmicutes* including *Lachnospiraceae CHKCI001* spp*.* and *Streptococcus sp002300045*. It is noteworthy that these clinically important ARGs encoding resistance to vancomycin (treating methicillin-resistant *staphylococcus aureus* (MSRA)) and rifamycin (treating tuberculosis) were only detected in an MGE-associated pattern (Fig. 4), and the high HGT potential implies the spread of clinical AMR from hospital to ambient air environments.

### Consumption of antibiotics and importance of β-lactam-resistant HAIs

The intensive use of antibiotics and the aerosolization of clinical waste reportedly contribute to the enrichment of airborne AMR in hospitals [27, 29]. Accordingly, ARGs in hospital PM_2.5_ were found to be significantly more abundant than in urban ambient air samples (*P* < 0.01; Fig. 1a). Table 1 shows β-lactams and fluoroquinolones as the predominantly used antibiotics (> 15,000 daily defined doses (DDDs)), and tetracyclines as the most intensively administered (~ 120 DDDs/patient). The data on their monthly average consumption varied significantly across all classes of antibiotic drugs without substantial seasonal changes (two-way ANOVA; antibiotic class: F = 158.5, *P* < 0.001; season: *P* = 0.67). This is consistent with the finding from a national survey that the (quarterly) amount of antibiotics consumed in China’s general hospitals had become stable in recent years due to improved antimicrobial stewardship [63]. There was no significant correlation between hospital PM_2.5_-ARGs and the overall corresponding usage of antibiotics in the hospital ward (Table 1). However, to some extent, the pattern of occurrence of PARB in air PM_2.5_ resembled variations in the intensity of antibiotic treatments, such as in the administration of β-lactams (broad-spectrum ones), glycopeptides, and tetracyclines on patients (*P* < 0.05; Table 1). This finding showed, for the first time, that the administration of antibiotics was partially related to the occurrence of PARB in airborne particles emitted from the hospital.

**Table 1:** Data on the administered antibiotics and their relationship with the PM2.5 resistome in the inpatient building

Antibiotic consumption		Agy	β-lactams			FQs	Gypt	MLs	TCs
		Aminoglycosides	Broad-spectrum	Carbapenems	Cephalosporins	Fluoroquinolones	Glycopeptides	Macrolides	Tetracyclines
^#^DDDs (monthly basis)	Summer	1740±280	4830±583	1680±73.3	13200±1310	15500±1330	365±55.8	8900±1100	2620±1100
			19700±746						
	Winter	1470±173	4350±176	1630±132	12600±748	17400±713	345±50.2	7910±1100	3050±541
			18600±549						
^#^DDDs/patient (monthly basis)	Summer	49.4±5.5	7.61±2.52	5.88±0.19	6.94±1.10	19.1±1.32	3.71±0.60	19.7±1.27	112±1.86
			6.92±1.95						
	Winter	50.7±3.5	8.10±0.21	5.87±0.73	7.48±0.44	19.10±0.38	3.49±0.45	23.3±3.01	136±28.3
			7.42±0.29						
Correlations with corresponding ARGs^†^		*P*_DDDs_ = 0.35 *P*_Intensity_ = 0.50	*P*_DDDs_ = 0.65 *P_Intensity_ = 0.02 (*R*_Intensity_ = − 0.90)	*P*_DDDs_ = 0.13 *P*_Intensity_ = 0.80	*P*_DDDs_ = 0.42 *P*_Intensity_ = 0.51	*P*_DDDs_ = 0.71 *P*_Intensity_ = 0.56	*P*_DDDs_ = 0.41 *P*_Intensity_ = 0.29	*P*_DDDs_ = 0.91 *P*_Intensity_= 0.71	*P*_DDDs_ = 0.56 *P*_Intensity_ = 0.42
			*P*_DDDs_ = 0.56; *P*_Intensity_=0.49						
Correlations with corresponding PARB^‡^		*P*_DDDs_ = 0.11 *P*_Intensity_ = 0.51	*P*_DDDs_ = 0.65 *P_Intensity_ = 0.0043 (*R*_Intensity_ = − 0.95)	*P*_DDDs_ = 0.25 *P*_Intensity_ = 0.79	*P*_DDDs_ = 0.41 *P*_Intensity_ = 0.47	*P_DDDs_ = 0.04 (*R*_DDDs_ = − 0.83) *P*_Intensity_ = 0.08	*P*_DDDs_ = 0.10 *P_Intensity_ = 0.05 (*R*_Intensity_ = 0.79)	*P*_DDDs_ = 0.32 *P*_Intensity_ = 0.39	*P*_DDDs_ = 0.31 *P_Intensity_ = 0.05 (*R*_Intensity_ = 0.81)
			*P*_DDDs_ = 0.48; *P*_Intensity_ = 0.07						

# Defined daily dose values (DDDs) representing consumption and antibiotic drugs were calculated according to the WHO guidelines; the intensity of the antibiotic treatments is shown by DDDs normalized to the number of patients to whom they were administered. Considering the limited size of the data (each season = 3), correlations were analyzed by covering the whole period of study, and significant ones were denominated with an asterisk and correlation coefficient values

† Data were retrieved from short-read mapping results, to represent the abundances of ARGs in hospital air PM_2.5_

‡ Data were retrieved from bacterial genome-binning results, to represent the contents of different types of potential antibiotic-resistant bacteria (PARB) in hospital air PM_2.5_

**Fig. 5:**
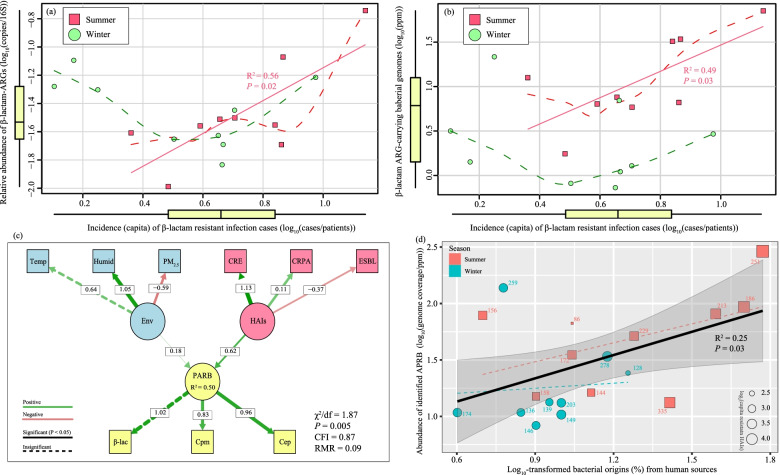
Clinical datasets regarding the number of cases of AMR healthcare-associated infections (HAIs) were collected and normalized to the number of patients on the sampling day to represent the incidence rate at the hospital’s Inpatient Department (HAIs/patient). Their correlations were analyzed with the relative abundance of ARGs (**a**), and that of ARG-hosting bacterial genomes (**b**). Data collected in summer and winter were depicted with red squares and green dots, respectively; solid lines indicate significant correlations and are annotated with a *P* value and a coefficient of correlation (*R*^2^). The dashed curves generally describe the trend in the variance of the datasets by locally estimated scatterplot smoothing (span = 0.75). **c** Structural equation modeling (SEM) analysis of the impacts of environmental factors (Env) and reported HAIs in wards on the variations of the abundance of carbapenem (Cpm), cephalosporin (Cep), and other β-lactam-resistant genes carrying bacterial genomes. **d** Linear correlations between the estimated percentages of bacterial origins from the human-source associated microbiome and the total abundance of potential antibiotic-resistant bacteria (PARB). The linear relationship fitting a significant correlation (Pearson, *P* < 0.05) is depicted using a solid line with confidential intervals (gray shades), while the insignificant relationship is shown using a dashed line. The sizes of the dots are in proportion to the log_2_-transformed HAIs that were recorded on the sampling day and the numbers of patients are indicated

The concentrations of ARG-carrying bacteria could also be affected by antibiotic-resistant HAIs (SI-Additional file 1: Table S3), given that human sources were considered the largest attributable sources to the bacterial community in the hospital-specific air PM_2.5_ (Fig. 1d). Although significant correlations were not detected between the total abundance of ARGs and the incidence of HAIs (HAIs/patient; *P* > 0.05; Additional file 1: Fig. S7), the incidence of β-lactam-resistant HAIs, which made up the majority of AMR cases (*n* = 1744 cases, Additional file 1: Table S3 and Fig. S8), was significantly correlated with the relative abundance of β-lactam ARGs (*R*^2^ = 0.56, *P* = 0.02; Fig. 5a) and their hosting bacteria (*R*^2^ = 0.49, *P* = 0.03; Fig. 5b) during the summer sampling time. Overall, β-lactam-resistant HAIs predominantly influenced the variations in the concentrations of β-lactam-resistant bacterial genomes in the PM_2.5_ samples over the environmental factor group (individual effects: 53% *vs.* 3%, Table S4). Specifically, as the most influential latent explanatory factor (SEM, std. = 0.62, Fig. 5c), the β-lactam-resistant HAIs, being positively structured by the carbapenem-resistant *Enterobacteriaceae* (CRE) and *Pseudomonas aeruginosa* (CRPA), significantly contributed to the varying occurrences of PM_2.5_-borne PARB carrying carbapenem- (Cpm, std. = 0.83) and cephalosporin- (Cep, std. = 0.96) resistant genes (*P* < 0.01, Fig. 5c). Compared to other HAI cases (Additional file 1: Fig. S8), the most prevalently detected carbapenem-resistant HAI cases (CRE) imposed the strongest effect on the distribution of genomic β-lactam ARGs in the hospital airborne PM_2.5_ (SI-Additional file 1: Fig. S9). Although the winter samples exhibited no significant correlations between β-lactam-resistant HAIs and β-lactam-related ARGs (Fig. 5a), bacterial taxa were still detected, including *Enterobacteriaceae Providencia* (8.16 genome copies/ppm reads) and *Staphylococcaceae Staphylococcus* (1.96 genome copies/ppm reads, Fig. 2b) hosting *bla*_OXA_ and *penA* encoding resistance to β-lactam antibiotics (SI-Additional file 1: Table S5). As such, the emitted airborne ARGs and PARB from the ventilation outfall of hospitals, which could stem from the aerosolization of sewage/moisture leakage [64], human skin, and respiration [65], remain an important AMR concern. Ventilation and air conditioning systems are heavily used in hospital wards during hot and humid summers in cities like Guangzhou. The higher relative humidity (Humid std. = 1.5, Fig. 5c) may favor the survival of airborne bacteria [64], as indicated by its significant correlation with the abundance of PARB metagenomes (Pearson, *P* < 0.01, Fig. S10). Both factors may lead to an increase in emissions of AMR from the studied hospital to the ambient air [27].

### AMR exposure hazards of hospital-influenced PM_2.5_ and risk rankings

This study shows that the PM_2.5_ emitted from the sampled hospital were efficient carriers of airborne ARGs and ARG-hosting bacteria, the compositions and occurrences of which were also closely linked to clinical activities (Fig 5c). According to a previous study conducted in Guangzhou city, the structures of the bacterial community in airborne particles were related more to the emission sources than to seasonal changes in weather. Similarly, our source tracing analysis estimated an intensive level (20~40%) of bacterial input from human-associated sources (Fig. 1d), which was significantly related to the abundance of PARB in hospital air PM_2.5_ (Fig. 5d). This “source-influenced airborne (PM_2.5_) AMR” relationship was therefore evaluated in the current study on a broader scale from exposure to potential risks (SI-Additional file 1: Table S6).

**Fig. 6:**
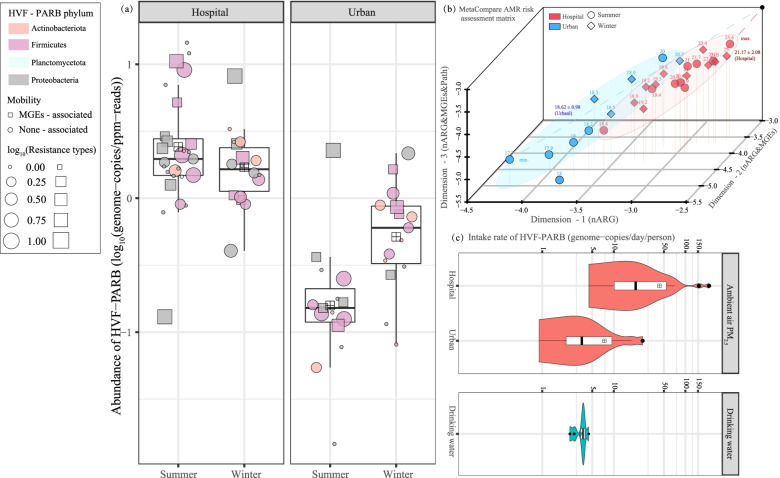
**a** Abundance of potential antibiotic-resistant bacteria (PARB) carrying human virulent factors (HVF) in hospital and urban PM_2.5_ samples in summer and winter. The HVF-PARB classified within the identical phylum are in the same color, and the MGE-associated genomic ARGs are presented in squares, the size of which are proportional to the number of resistance types of ARGs hosted by the HVF-PARB. **b** The AMR risk scores were estimated using MetaCompare. The *x*, *y*, and *z* axes represent the portions of contigs concerning the ARGs, MGE-associated ARGs, and pathogen-hosting ARGs to the total assembled contigs. The black dot (vertex) indicates the theoretically highest AMR risk, and the relative-risk score of each sample is labeled. The red and blue dots indicate the hospital and urban samples, respectively. The summer and winter samples are represented by circles and squares, respectively. **c** Intake rate of the HVF-PARB from urban and hospital-influenced air particulates (PM_2.5_) and drinking water. The quantification of the concentrations and corresponding calculations of the target bacteria genomes were based on the relative abundance of the non-redundant metagenomes in each sample (SI-3)

The HVF-PARB were primarily classified as *Actinobacteria*, *Proteobacteria*, and *Firmicutes* (Fig. 6a), and no significant differences were noted in their abundance across the bacterial taxa (one-way ANOVA, *P* = 0.77). However, these HVF-PARB metagenomes in the hospital samples were nearly one order of magnitude higher in abundance (0.28 ± 0.23 log_10_(genome copies/ppm reads)) than in urban ambient air (*T*-test, *P* < 0.001), especially during the summer (Fig. 6a). As to the HVF-PARB that carried potentially mobile ARGs, they possessed significantly more resistant types (5.00 ± 3.07 *vs.* 3.01 ± 1.77, Fig. 6a) in the hospital-specific PM_2.5_ (one-way ANOVA, F = 11.0, *P* < 0.01) than in the urban (sampled in Guangzhou) ambient air PM_2.5_ (one-way ANOVA, *P* = 0.13). This finding implies that ARGs were more prone to dissemination across the HVF-PARB via the MGE-mediated HGT process in the hospital airborne PM_2.5_ than in the relevant urban (non-clinical) sources. More importantly, the HVF-PARB hosting mobile (MGEs-associated) ARGs were ranked as being of the highest AMR risk concern [66]. As shown in Fig. 6b, by using MetaCompare [47], the highest risk was identified in hospital PM_2.5_ as 25.60, which is the closest distance to the theoretical maximum AMR vertex in the risk ranking matrix, whereas the lowest score was found in the urban samples (17.70). Overall, the mean risk index score of the hospital PM_2.5_ (21.17 ± 2.08) was significantly higher than that of urban ones, but none of them exhibited seasonal differences (two-way ANOVA, F_site_ = 12.7, *P*_site_ < 0.01, *P*_season_ = 0.79). This indicates that antibiotic resistomes in the hospital samples were featured with higher abundance and HGT mobility and were hosted more by human pathogens than in urban air PM_2.5_ samples. This lower risk ranking index value of the collected urban air PM_2.5_ samples was probably caused by the lower input of AMR than from source-impacted locations [25, 30, 67], which may be partially explained by the lower human-associated bacteria input (Fig. 1d), and/or by dilution through aerial transport from the sources of emission [68]. It should be noted that the AMR index merely differentiated all selected sites by a score of 3.5, but ARGs in hospital air PM_2.5_ more frequently co-occurred with MGEs and HVFs (*z*-axis values; *T*-test, *P* < 0.01, Fig. 6b). This suggests that improvements need to be made to the resistome risk ranking method to more clearly label the AMR health hazards to human beings.

To highlight the importance of PARB and to further explore their AMR risks, we calculated the concentrations of HFV-PARB that are pathogenic to human beings. The mean concentration in the hospital samples was 2.89 ± 3.64 genome copies/m^3^-air (*n* = 19), which was around five times higher than in the urban ambient air PM_2.5_ (*n* = 10, SI-Additional file 1: Table S7). This difference became more pronounced with the HVF-PARB that harbored mobile (MGE-associated) ARGs; there were an average of 1.12 ± 1.81 genome copies/m^3^-air in the hospital PM_2.5_ samples in comparison to 0.18 ± 0.12 genome copies/m^3^-air in the urban ambient air samples (Kruskal-Wallis test, *χ*^2^ = 11.2, *P* = 0.001, *n* = 29). As such, an adult would inhale ~ 45 genome copies of HFV-PARB daily from the ambient air PM_2.5_ emitted from the sampled hospital (15 m^3^/day [10]; Fig. 6c). This is generally consistent with the results of resistome risk estimations (Fig. 6c), which indicate that the (Guangzhou) urban air PM_2.5_ had a lower AMR ranking index value than the hospital-emitted ones. Apart from inhalation, the ingestion of drinking water is another direct pathway of human exposure (2 L/day) to environmentally disseminated AMR [69, 70]. Of particular interest is the finding that drinking water contained 1.42 ± 0.28 genome copies/L of HVF-PARB (Additional file 1: Table S7). Hence, the daily intake of virulent antibiotic-resistant bacteria via drinking water was 3.55 ± 0.61 genome copies/day (*n* = 6; Fig. 6b), which was nearly 10 times lower than that from the inhaled air PM_2.5_ in the study (Kruskal-Wallis test, *χ*^2^ = 19.55, *P* < 0.001; *n* = 35). From the perspective of “One Health” [5], this finding further suggests that the inhalation of air PM_2.5_, particularly that emitted from hospitals, may be an important AMR exposure pathway to human beings. However, due to the limitations regarding the geographical distribution of the PM_2.5_ samples collected from Guangzhou city (Southern China), a more holistic study remains to be conducted on the AMR hazards and risk rankings of source-specificair PM_2.5_.

## Conclusions

The present study revealed a genome-resolved “panorama” of antibiotic resistomes in airborne PM_2.5_ in a typical municipal hospital and in urban ambient air. The hospital-specific resistome was significantly correlated with the dynamically varied structures of the bacterial community (Procrustes test; permutations = 999, *M*^2^ = 0.39, *P* < 0.001)), to which the human-associated microbiome (~ 30%)  was the largest contributor. The patterns of occurrence of ARG-carrying bacteria in hospital airborne PM_2.5_ were potentially influenced by the incidence of β-lactam HAIs in wards, highlighting a close relevance to the spread of AMR via PM_2.5_ from clinical sources to the surrounding air environments, especially in summer (Pearson; *P* < 0.05) when more precautions should be taken in air ventilation disinfection procedures. Compared to urban ambient air samples, the higher abundance (twofold, *P* = 0.05), diversity, and mobility of ARGs carried by potentially virulent bacteria in hospital-specific PM_2.5_ (2.89 ± 3.64 genome copies/m^3^-air) led to significantly more AMR hazards (MetaCompare index = 21.17 ± 2.08, *P* < 0.01), to which humans could be exposed. It should be noted that the current study collected samples confining to one city in China. For better generalization of site-specific AMR features, comparative studies of airborne antibiotic resistomes and risk assessments encompassing hospitals (clinical) and other anthropogenic sources (e.g., landfills and wastewater treatment plants) across different geographical locations would be required. Our findings suggested that ARG-carrying bacteria in hospital airborne PM_2.5_ were potentially influenced by the incidence of HAIs in wards, while this AMR transmission chain (source – air – community) has yet to be systematically identified. As such, culture-based studies of source specific airborne particles, especially on a larger geographical scale, are warranted to further examine the airborne-resistant pathogens associated with hospitals and the chains linking them to the development of AMR in the surrounding urban communities. Based on estimations of the intake of AMR materials, differences concerning human immunological responses to AMR exposure (respiration *vs.* digestion systems) should also be included in future risk assessments, particularly to compare multiple exposure pathways from the “One Health” perspective.

## Supplementary information

**Additional file 1: **Supporting Information. **SI-1.** On-site sampling and additional information on samples: **Fig. S1.** Installation of the on-site sampler (**a**) and the collected microfiber filter (**b**). **Table S1.** Sampling information and DNA concentrations. **Table S2.** Air quality information on the sampling days. **SI-2.** Information related to ARGs and the bacterial community: **Fig. S2.** Relative abundance of antibiotic resistomes in hospital and urban PM_2.5_ samples. **Fig. S3.** Relative abundance of the profiled microbes (Phylum-level) in the collected samples. **Fig. S4.** Relative abundance of the profiled microbes (Class-level) in the collected samples. **Fig. S5.** Linear discriminant analysis Effect Size (LEfSe) of the bacterial taxa in all PM_2.5_ samples (the effects of seasonal differences were blocked. **Fig. S6.** Linear discriminant analysis Effect Size (LEfSe) of the bacterial taxa in all PM_2.5_ samples (the effects of site differences were blocked). **Fig. S7.** Correlations between the identified AMR infection incident rate and the relative abundance of total PM_2.5_-ARGs hosted in hospital air PM_2.5_ samples. The cases of resistant HAIs were analyzed on a daily (**a**) and weekly basis (**b**). The dashed curves generally describe the trend in the variance of the datasets by locally estimated scatterplot smoothing (span = 0.75). **Fig. S8.** The number of detected cases of AMR infections in the hospital (inpatient department-HAIs). **Table S3.** Data on the infection cases collected from the inpatient department**. SI-3.** Supplementary information on statistics and sequencing data sources: **Fig. S9.** Relative influence of variables in the groups of (β-lactam) AMR infections. **Fig. S10.** Correlations between identified PARB in hospital-specific PM_2.5_ and the relative humidity in the ambient metagenomes. **Table S4.**Variation partitioning analysis (VPA) table. **Table S5.** Taxonomic classification of the assembled potential β-lactam resistant metagenomes. **Table S6.** Specific information on the retrieved metagenomic sequencing data. **Table S7.** Mean concentrations of the targeted potentially virulent antibiotic-resistant bacteria genomes in airborne PM_2.5_ and drinking water.

**Additional file 2.** Supporting data list 1. Compositions and relative abundance of the resistomes in hospital and urban air PM_2.5_ samples.

**Additional file 3.** Supporting data list 2. Compositions and relative abundance of the bacterial community in hospital and urban air PM_2.5_ samples.

**Additional file 4.** Processing pipelines of sequencing data.

## Data Availability

The sequencing data generated from Illumina metagenomes in this study have been deposited with the accession number PRJNA726763. The details and accession numbers of the retrieved sequencing datasets are provided in the Supplementary Information (Additional file 1: Table S5). Other data or processing pipelines from this study are available in the Supplementary Information (Additional File 4).

## Abbreviations

ARG: Antibiotic-resistant gene
AMR: Antimicrobial resistance
HAI: Healthcare-associated infection
CRE: Carbapenem-resistant *Enterobacteriaceae*
HGT: Horizontal gene transfer
HVF: Human virulent factor
MGE: Mobile genetic element
ORFs: Open reading frames
PARB: Potential antibiotic-resistant bacteria
RH: Relative humidity
WHO: World Health Organization
