# Supplementary material for: Inhalable antibiotic resistomes emitted from hospitals: metagenomic insights into bacterial hosts, clinical relevance, and environmental risks
Source: Microbiome. 2022 Jan 27;10:19. doi: 10.1186/s40168-021-01197-5 (PMC8796446; doi:10.1186/s40168-021-01197-5)
Supplement: Supplementary file 2 — Additional file 1: Supporting Information. SI-1. On-site sampling and additional information on samples: Fig. S1. Installation of the on-site sampler (a) and the collected microfiber filter (b). Table S1. Sampling information and DNA concentrations. Table S2. Air quality information on the sampling days. SI-2. Information related to ARGs and the bacterial community: Fig. S2. Relative abundance of antibiotic resistomes in hospital and urban PM2.5 samples. Fig. S3. Relative abundance of the profiled microbes (Phylum-level) in the collected samples. Fig. S4. Relative abundance of the profiled microbes (Class-level) in the collected samples. Fig. S5. Linear discriminant analysis Effect Size (LEfSe) of the bacterial taxa in all PM2.5 samples (the effects of seasonal differences were blocked. Fig. S6. Linear discriminant analysis Effect Size (LEfSe) of the bacterial taxa in all PM2.5 samples (the effects of site differences were blocked). Fig. S7. Correlations between the identified AMR infection incident rate and the relative abundance of total PM2.5-ARGs hosted in hospital air PM2.5 samples. The cases of resistant HAIs were analyzed on a daily (a) and weekly basis (b). The dashed curves generally describe the trend in the variance of the datasets by locally estimated scatterplot smoothing (span = 0.75). Fig. S8. The number of detected cases of AMR infections in the hospital (inpatient department-HAIs). Table S3. Data on the infection cases collected from the inpatient department. SI-3. Supplementary information on statistics and sequencing data sources: Fig. S9. Relative influence of variables in the groups of (β-lactam) AMR infections. Fig. S10. Correlations between identified PARB in hospital-specific PM2.5 and the relative humidity in the ambient metagenomes. Table S4.Variation partitioning analysis (VPA) table. Table S5. Taxonomic classification of the assembled potential β-lactam resistant metagenomes. Table S6. Specific information on the retrieved metagenomic seq [file 40168_2021_1197_MOESM1_ESM.docx]

**Supporting Information**

**Inhalable antibiotic resistome emitted from hospitals: Metagenomic insights into bacterial hosts, clinical relevance and environmental risks**

Dong Wu^1,2^, Ling Jin^1,3^, Jiawen Xie^1^, Hang Liu^4^, Jue Zhao^1^, Dan Ye^5*^, and Xiang-dong Li^1*^

^1^ *Department of Civil and Environmental Engineering, The Hong Kong Polytechnic University, Kowloon, Hong Kong SAR, China*

^2^ *Shanghai Engineering Research Center of Biotransformation of Organic Solid Waste, School of Ecological and Environmental Science, East China Normal University, Shanghai 200241, China*

^3^ *Department of Health Technology and Informatics, The Hong Kong Polytechnic University, Kowloon, Hong Kong SAR, China*

^4^ *University Research Facility in Chemical and Environmental Analysis, The Hong Kong Polytechnic University, Kowloon, Hong Kong SAR, China*

*^5^ The First Affiliated Hospital of Guangzhou Medical University, Guangzhou 440104, China*

Dong Wu (dwu@des.ecnu.edu.cn)

Ling Jin (ling.jin@polyu.edu.hk)

Jiawen Xie (jiawen.xie@connect.polyu.hk)

Hang Liu (hang.liu@polyu.edu.hk)

Jue Zhao (jue2018.zhao@connect.polyu.hk)

*Corresponding author: Prof. Xiang-dong Li

Address: Department of Civil and Environmental Engineering, The Hong Kong Polytechnic University, Hung Hom, Kowloon, Hong Kong SAR, China

Email: [cexdli@polyu.edu.hk](mailto:cexdli@polyu.edu.hk)

Phone: (852) 2766-6041

Fax: (852) 2334-6389

ORCID: 0000-0002-4044-2888

*Co-corresponding author: Dr. Dan Ye

Address: 151 West Yanjiang Rd., The First Affiliated Hospital of Guangzhou Medical University, Guangzhou, Guangdong 440104, China

Email: [yedan@gyfyy.com](mailto:yedan@gyfyy.com)

# SI-1 On-site sampling and information on the samples

The sampling site was located on the ventilation outfalls on the top of the inpatient building of a 1,500-bed urban general hospital in Guangzhou that serves more than 150,000 inpatients annually. A large-volume sampler (ASM-1, Mingye Inc. China) was set at a flow rate of 1.0 m^3^/min to collect the fine particles (PM_2.5_) emitted from the inpatient (Inpatient Department) building (**Fig. S1a**). The hospital-PM_2.5_ was selected from total airborne particles by cyclone separation and then was deposited on a preheated (500 °C for 5 h) quartz microfiber filter (A4 size, PALL Life-Science, US).


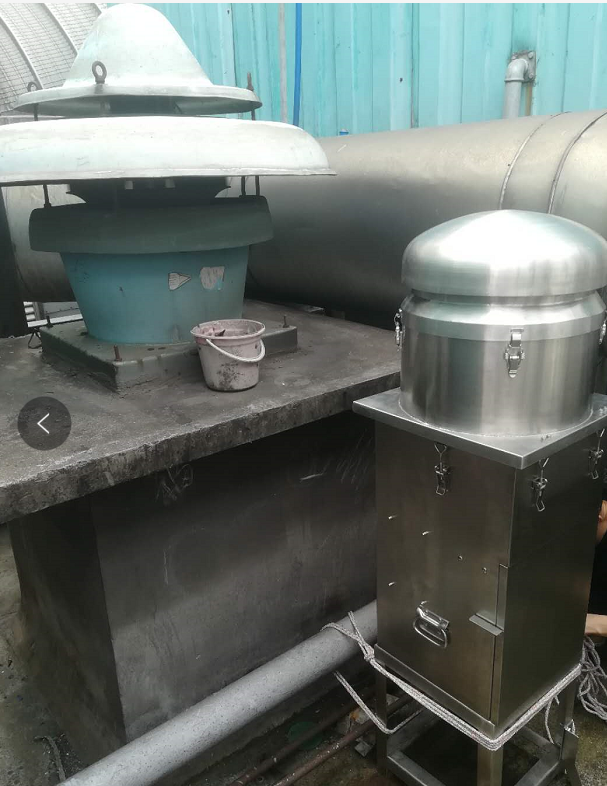

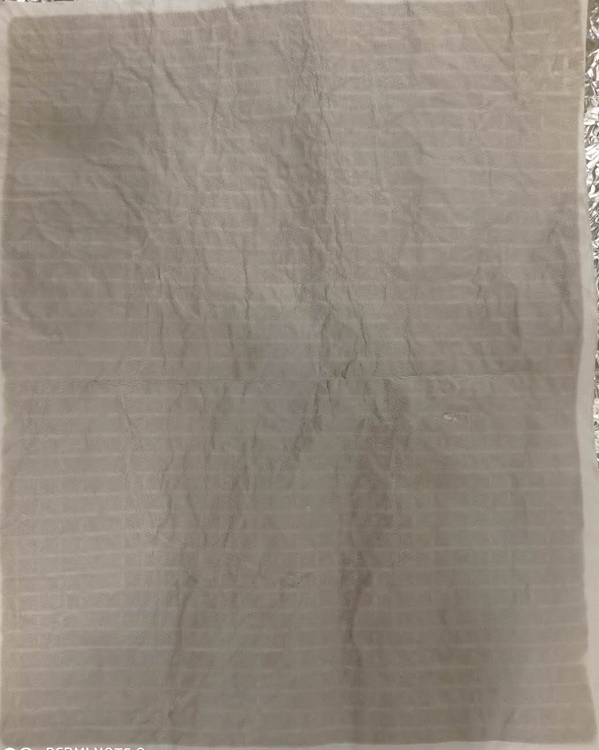


**Fig. S1** Installation of the on-site air PM_2.5_ sampler (**a**); and the collected microfiber filter (**b**)

The sampling campaign was conducted from June to December 2019, which covered the summer and winter seasons in Guangzhou. In each month, two to three samples were collected at the sampling site. Each sample was collected based on a 24 h sampling time (**Fig. S1b**), and a total of 25 samples were collected during the sampling period. All filter samples were stored at -20 ^o^C before the pretreatment process. In addition, to conduct a comparative analysis of hospital-specific samples in the same city (**Table S1**), urban ambient air PM_2.5_ samples collected from Guangzhou (Tianhe (TH) and Chonghua (CH) Districts) from April of 2016 to May of 2017 were used in this study. Due to the limited sizes of preserved filter samples in the lab, samples collected from late April to August were combined for DNA extraction (Summer season), while samples collected from late September to February were combined for DNA extraction (Winter season).

Half of the A4-size filter was cut out using a pair of disinfected scissors. The pretreatment process generally followed the published protocol with several modifications [1]. The cut-out filter was evenly shredded into three pieces, each of which was sonicated with sterilized 1× phosphate-buffered saline (~ 40 mL) for 20 minutes in a centrifugal tube. This sonication procedure was repeated three times, during which the PBS-extract aliquots from the same sample were combined, and then were filtered through a PES membrane disc-filter (0.2-μm × 47 mm, Supor 200, PALL Co., US). The obtained disc-filters were stored at -20 ℃ before use.

The metagenomic DNA of each PM_2.5_ sample was extracted from a whole disc-filter by using a FastDNA Spin Kit (MP, USA). The disc-filter was shredded at first and then was incubated with Lysing Matrix E (MP, USA) at 65 ℃ for 15 min. The mixture was then vortexed for 15 min on a homogenizer (Genie 2, Scientific Industries, USA) to lyse cell membranes. The resulting solutions were purified by using Protein Precipitation Solution (PPS) according to manufactures’ instructions. An Agencourt AMPure XP bead kit was used to improve the yield of DNA (Beckman Coulter, USA). The extracted metagenomic DNA was verified by agarose gel electrophoresis. The DNA yields were determined by a dsDNA-binding dye assay on the Qubit3.0 Fluorometer (ThermoFisher Scientific, US, Table S1). All of the DNA extracts were kept at −80°C until they were analyzed.

**Table S1** Sampling information and DNA concentrations

| Name | Sequencing-size (Gigabytes) | Concentration (ng/μL) | Site - season | Date (YYYY/MM/DD) |
| --- | --- | --- | --- | --- |
| GZ1 | 6.32 Gb ×2 | 0.3 ng/μL | Hospital summer | 20190619 |
| GZ2 | 6.39 Gb ×2 | 0.6 ng/μL | Hospital summer | 20190624 |
| GZ3 | 6.32 Gb ×2 | 0.5 ng/μL | Hospital summer | 20190627 |
| GZ4 | 6.45 Gb ×2 | 0.8 ng/μL | Hospital summer | 20190710 |
| GZ5 | 6.43 Gb ×2 | 0.6 ng/μL | Hospital summer | 20190718 |
| GZ6 | 6.40 Gb ×2 | 1.3 ng/μL | Hospital summer | 20190725 |
| GZ7 | 6.25 Gb ×2 | 0.5 ng/μL | Hospital summer | 20190803 |
| GZ8 | 6.17 Gb ×2 | 0.3 ng/μL | Hospital summer | 20190812 |
| GZ9 | 6.39 Gb ×2 | 0.2 ng/μL | Hospital summer | 20190823 |
| GZ10 | 6.30 Gb ×2 | 0.3 ng/μL | Hospital summer | 20190827 |
| GZ12 | 8.33 Gb ×2 | 0.9 ng/μL | Hospital winter | 20190925 |
| GZ13 | 8.35 Gb ×2 | 0.4 ng/μL | Hospital winter | 20191016 |
| GZ14 | 8.38 Gb ×2 | 1.0 ng/μL | Hospital winter | 20191104 |
| GZ15 | 8.31 Gb ×2 | 0.8 ng/μL | Hospital winter | 20191111 |
| GZ16 | 8.41 Gb ×2 | 0.7 ng/μL | Hospital winter | 20191114 |
| GZ21 | 8.12 Gb ×2 | 0.2 ng/μL | Hospital winter | 20191128 |
| GZ22 | 8.33 Gb ×2 | 0.5 ng/μL | Hospital winter | 20191205 |
| GZ24 | 8.35 Gb ×2 | 0.9 ng/μL | Hospital winter | 20191223 |
| GZ25 | 8.14 Gb ×2 | 0.4 ng/μL | Hospital winter | 20191225 |
| CH1 | 8.55 Gb ×2 | 0.3 ng/μL | Urban summer | 201604 to 201605 |
| CH2 | 8.13 Gb ×2 | 0.2 ng/μL | Urban summer | 201606 to 201608 |
| CH5 | 8.43 Gb ×2 | 0.2 ng/μL | Urban summer | 201704 to 201705 |
| TH1 | 7.50 Gb ×2 | 0.1 ng/μL | Urban summer | 201604 to 201605 |
| TH2 | 7.95 Gb ×2 | 0.2 ng/μL | Urban summer | 201606 to 201608 |
| TH5 | 8.11 Gb ×2 | 0.3 ng/μL | Urban summer | 201704 to 201705 |
| CH3 | 8.22 Gb ×2 | 0.1 ng/μL | Urban winter | 201609 to 201612 |
| CH4 | 7.54 Gb ×2 | 0.2 ng/μL | Urban winter | 201701 to 201702 |
| TH3 | 8.26 Gb ×2 | 0.2 ng/μL | Urban winter | 201609 to 201612 |
| TH4 | 8.30 Gb ×2 | 0.1 ng/μL | Urban winter | 201701 to 201702 |

Information on the air quality on the sampling day was retrieved from an online platform (**Table S2**), the data of which were recorded by a municipal real-time monitoring system. Our hospital air PM_2.5_ sampling site was 1.2 kilometers north of this monitoring station (Shi Wu Zhong, Guangzhou City). The hospital and the air quality monitoring station were both located in the same district in Guangzhou City.

**Table S2** Air quality information on the sampling days

| *Date  (YYYY/MM/DD) | Temp  (℃) | Humidity  (%) | Wind (scale) | AQI | PM_2.5_  (μg/m³) | PM_10_  (μg/m³) | SO_2_  (μg/m³) | CO  (mg/m³) | NO_2_  (μg/m³) | O_3_  (μg/m³) |
| --- | --- | --- | --- | --- | --- | --- | --- | --- | --- | --- |
| 20190619 | 28 | 87 | 0 | 49 | 14 | 28 | 5 | 0.7 | 39 | 87 |
| 20190624 | 25 | 93 | 0 | 55 | 14 | 23 | 5 | 0.8 | 44 | 26 |
| 20190627 | 27 | 86 | 0 | 69 | 18 | 36 | 5 | 0.7 | 38 | 153 |
| 20190710 | 29 | 83 | 1 | 52 | 13 | 20 | 5 | 0.7 | 41 | 25 |
| 20190718 | 31 | 67 | 0 | 70 | 36 | 62 | 8 | 0.8 | 40 | 124 |
| 20190725 | 29 | 78 | 1 | 65 | 15 | 32 | 6 | 0.6 | 31 | 117 |
| 20190803 | 25 | 88 | 0 | 38 | 18 | 36 | 6 | 0.6 | 32 | 139 |
| 20190812 | 30 | 70 | 0 | 96 | 22 | 37 | 7 | 0.7 | 26 | 144 |
| 20190823 | 30 | 76 | 1 | 120 | 25 | 47 | 8 | 0.7 | 37 | 168 |
| 20190827 | 30 | 76 | 1 | 120 | 25 | 47 | 8 | 0.7 | 37 | 168 |
| 20190925 | 25 | 46 | 2 | 50 | 25 | 51 | 7 | 0 | 36 | 98 |
| 20191016 | 25 | 63 | 1 | 45 | 29 | 45 | 7 | 0 | 32 | 72 |
| 20191104 | 23 | 41 | 2 | 66 | 48 | 90 | 10 | 0 | 28 | 139 |
| 20191111 | 21 | 53 | 0 | 88 | 58 | 110 | 12 | 0 | 8 | 82 |
| 20191114 | 20 | 54 | 1 | 59 | 39 | 68 | 7 | 0 | 37 | 91 |
| 20191128 | 16 | 53 | 2 | 36 | 15 | 36 | 8 | 0 | 30 | 31 |
| 20191205 | 14 | 32 | 3 | 51 | 26 | 53 | 10 | 0 | 35 | 68 |
| 20191223 | 16 | 52 | 1 | 66 | 42 | 81 | 10 | 1 | 61 | 30 |
| 20191225 | 14 | 57 | 0 | 72 | 44 | 94 | 8 | 1 | 78 | 38 |

*Data sources: <https://www.aqistudy.cn/> (Chinese); <https://aqicn.org/city/guangzhou/> (English)

# SI-2 Information related to ARGs and the bacterial community in air samples

The relative abundances of antibiotic resistance genes (ARGs) were identified by the local alignment against the DeepARG database [2]. A total of 18 resistant types were detected (identity > 85% of 16S rRNA reference sequences, ARGs: identity > 80%, probability > 0.8). The major types included multidrug, macrolide–lincosamide–streptogramin (MLS), aminoglycoside, tetracycline, beta-lactam (β-lactam), bacitracin, rifamycin, sulfonamide, glycopeptide, peptide, and fluoroquinolone. They accounted for 99.9% of the total antibiotic resistance (resistome). **Fig. S2** shows the summed abundance of PM_2.5_-borne antibiotic resistome in both hospital and urban ambient air. Compared to the urban ambient air resistome, hospital samples (sum of the identified different types of ARGs) harbored a significantly more abundant resistome (0.19 ± 0.1 log_10_(ARGs/16S rRNA gene), which was about 0.2 - 0.5 orders of magnitude higher with statistical significance (One-way ANOVA, *P* = 0.05). It is interesting to note that the more abundant antibiotic resistome occurred in summer for both hospital and urban PM samples, but no statistical significance was detected (One-way ANOVA, *P* = 0.34).


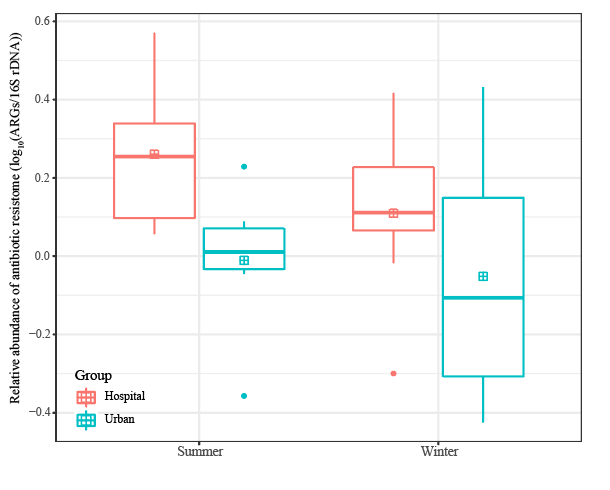


**Fig. S2** Relative abundance of antibiotic resistomes in hospital and urban PM_2.5_ samples


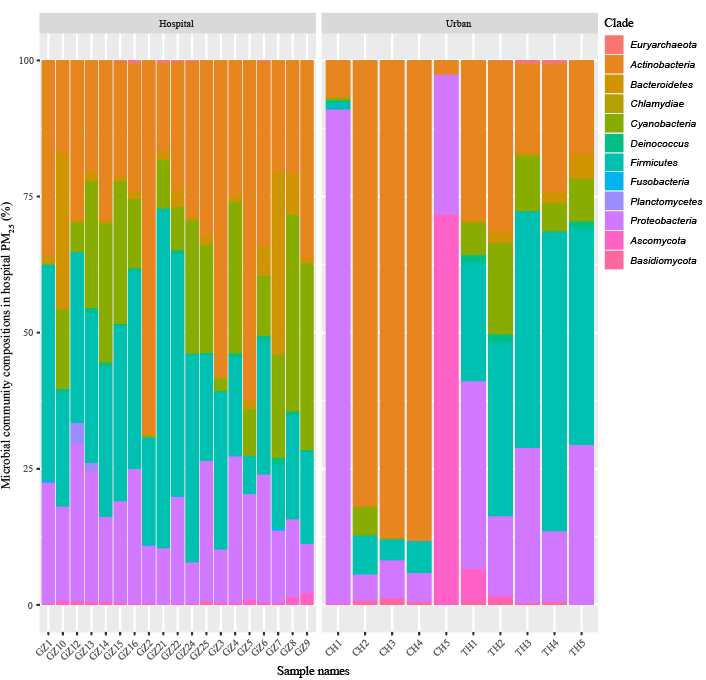
 **Fig. S3** Relative abundance of the profiled microbes (Phylum-level) in the collected air PM_2.5_ samples

The microbial community was profiled using metagenomic sequencing. The relative abundance of each phylum was shown in **Fig. S3**. The reads numbers of each detected bacterial taxon were normalized to the reads numbers that were mapped to 16S rRNA genes using MetaPhlAn3 (v3.0.6). There were a total of 13 bacterial phyla in the air PM_2.5_ samples. Among them, *Actinobacteria, Firmicutes, Cyanobacteria*, and *Proteobacteria* were the predominant phyla. There was a trend of decrease in *Actinobacteria* from summer to winter, while the portions of *Firmicutes* generally increased from 10% to 20%. At the same time, the relative abundances of *Proteobacteria* and *Cyanobacteria* remained similar. Notably, regarding the urban samples, more dynamic variations of the bacterial phyla were observed (**Fig. S3**). Among them, the TH samples that were collected from more downtown areas exhibited a more stable bacterial community structure than the CH ones, which features a substantially high abundance of *Proteobacteria* in summertime (~ 90%, CH1).


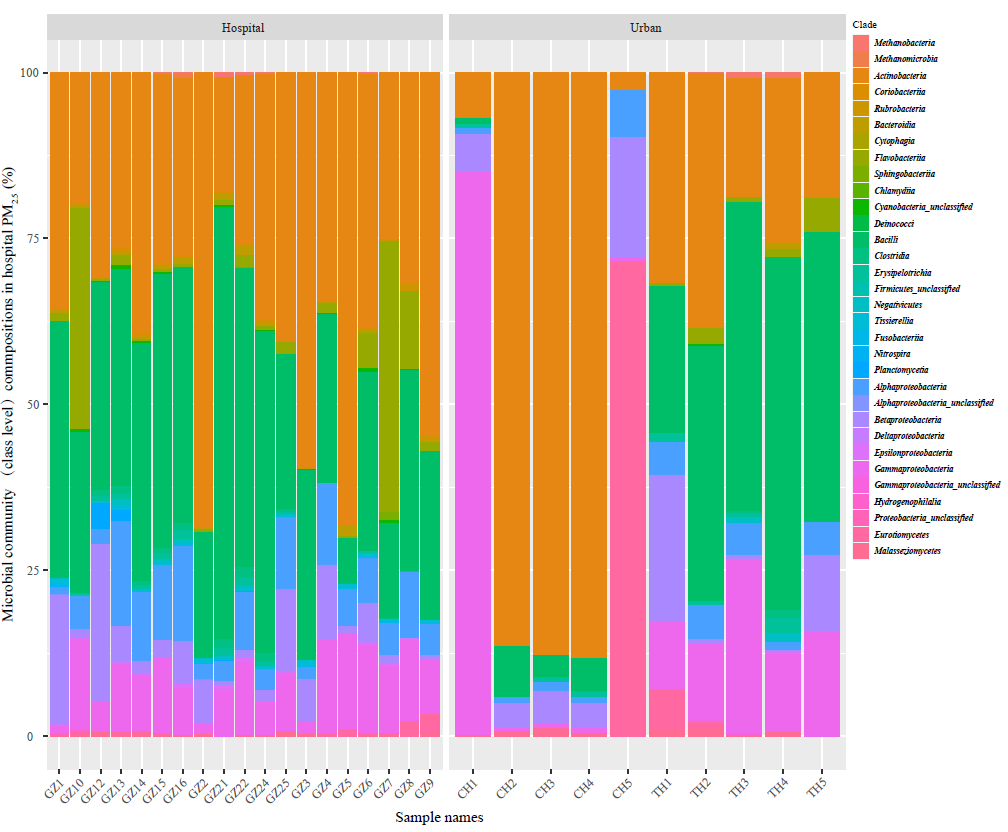


**Fig. S4** Relative abundance of the profiled microbes (Class-level) in the collected air PM_2.5_ samples

Microbial communities at the class level are shown in **Fig. S4**. Twenty-eight classes of bacteria were identified, most of which belonged to dominant phyla groups (**Fig. S3**). Their abundance was normalized to the number of 16S rRNA genes for cross-sample comparisons. The metagenomic taxonomic information of the bacterial community was analyzed from the paired-end clean reads of each sample. The predominant class of the bacterial phylum of *Actinobacteria* was identified as *Actinobacteria* (**Fig. S4**). *Bacilli* were the most abundant subtaxon class of *Firmicutes* (**Fig. S4**). *Proteobacteria* was primarily comprised of *Alpha*/*Gamma-Proteobacteria*. The relative abundance of *Gama-Proteobacteria* exhibited a trend of increase from summer to winter (*P* < 0.05), while that of the *Alpha-Proteobacteria* generally varied from 2% to 10%.


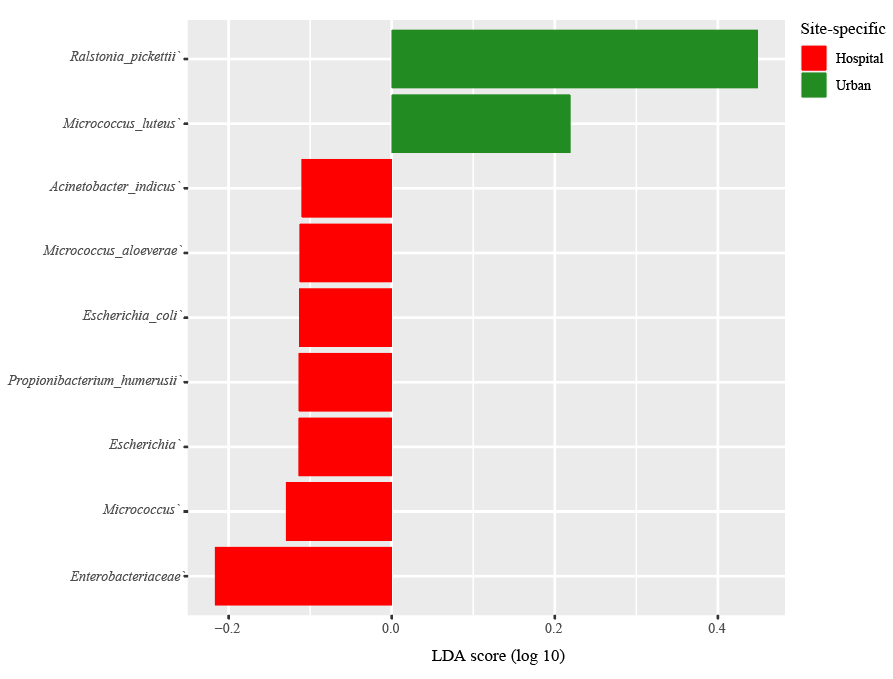


**Fig. S5** Linear discriminant analysis Effect Size (LEfSe) of the bacterial taxa in all PM_2.5_ samples (the effects of seasonal difference was blocked)

The MetaPhlAn outputs including the hospital and urban specific samples were compiled together to perform the Linear discriminant analysis Effect Size (LEfSe). The bacterial taxa that significantly contributed to variations of the whole bacterial community were identified with a log_10_-transformed LDA score larger than 0.1. As shown in **Fig. S5**, more bacteria in hospital samples were identified as the variation-dominated taxa than in the urban ones, which only included the *Ralstonia picketti* and *Micrococcus luteus*.

In addition, the hospital samples harbored more human-associated bacteria like *Es*cherichia coli, *Micrococcus* and *Enterobacteriaceae*. This is consistent with the source tracking analysis, which shows higher bacterial input from human wastes sources in the hospital-specific PM_2.5_. Apart from that, LEfSe analysis was conducted based on the changes of season. **Fig. S6** shows that more bacterial taxa influenced the bacterial community changes from summer to winter. In the wintertime, *Firmicutes* and their subtaxon bacteria were identified to more influential than others. Additionally, in the summer samples, most of the variation-contributing bacteria are more human-waste associated, such as *Corynebacterium spp*. and *Staphylococcus spp*. , both of which are human commensals and are usually implicated in human pathogenities.


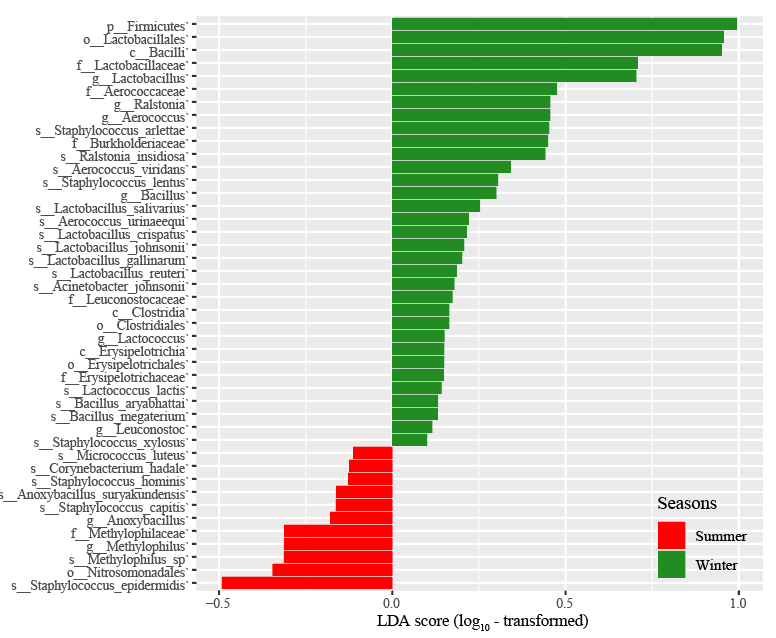


**Fig. S6** Linear discriminant analysis Effect Size (LEfSe) of the bacterial taxa in all PM_2.5_ samples (the effects of site difference was blocked)

**Table S3** Data on the AMR infection cases collected from the in-patient department

| **Sample** | **Total cases** | **Number of patients** | **Seasons** | **ARGs** | **Type** | **Cases** | **Portion%** |
| --- | --- | --- | --- | --- | --- | --- | --- |
| GZ1 | 251 | 12 | Summer | 0.180964 | beta-lactam | 166 | 66.1 |
| GZ10 | 86 | 17 | Summer | 0.024656 | beta-lactam | 39 | 45.3 |
| GZ12 | 174 | 18 | Winter | 0.01466 | beta-lactam | 83 | 47.7 |
| GZ13 | 146 | 17 | Winter | 0.020386 | beta-lactam | 79 | 54.1 |
| GZ14 | 203 | 21 | Winter | 0.023614 | beta-lactam | 94 | 46.3 |
| GZ15 | 136 | 16 | Winter | 0.022269 | beta-lactam | 51 | 37.5 |
| GZ16 | 149 | 14 | Winter | 0.035669 | beta-lactam | 71 | 47.7 |
| GZ2 | 186 | 12 | Summer | 0.028034 | beta-lactam | 83 | 44.6 |
| GZ21 | 128 | 23 | Winter | 0.080453 | beta-lactam | 34 | 26.6 |
| GZ22 | 278 | 22 | Winter | 0.052578 | beta-lactam | 28 | 10.1 |
| GZ24 | 139 | 18 | Winter | 0.060891 | beta-lactam | 170 | 122 |
| GZ25 | 259 | 27 | Winter | 0.049774 | beta-lactam | 48 | 18.5 |
| GZ3 | 213 | 17 | Summer | 0.084949 | beta-lactam | 125 | 58.7 |
| GZ4 | 172 | 19 | Summer | 0.020322 | beta-lactam | 138 | 80.2 |
| GZ5 | 335 | 24 | Summer | 0.006832 | beta-lactam | 201 | 60.0 |
| GZ6 | 229 | 24 | Summer | 0.031476 | beta-lactam | 122 | 53.3 |
| GZ7 | 156 | 17 | Summer | 0.030828 | beta-lactam | 77 | 49.4 |
| GZ8 | 158 | 20 | Summer | 0.010279 | beta-lactam | 61 | 38.6 |
| GZ9 | 144 | 19 | Summer | 0.027618 | beta-lactam | 74 | 51.4 |
| GZ1 | 251 | 12 | Summer | 0.646474 | multidrug | 41 | 16.3 |
| GZ10 | 86 | 17 | Summer | 0.234695 | multidrug | 20 | 23.3 |
| GZ12 | 174 | 18 | Winter | 0.037599 | multidrug | 45 | 25.9 |
| GZ13 | 146 | 17 | Winter | 0.20496 | multidrug | 28 | 19.2 |
| GZ14 | 203 | 21 | Winter | 0.245414 | multidrug | 44 | 21.7 |
| GZ15 | 136 | 16 | Winter | 0.060796 | multidrug | 26 | 19.1 |
| GZ16 | 149 | 14 | Winter | 0.316182 | multidrug | 47 | 31.5 |
| GZ2 | 186 | 12 | Summer | 0.263889 | multidrug | 62 | 33.3 |
| GZ21 | 129 | 20 | Winter | 0.21753 | multidrug | 45 | 34.9 |
| GZ22 | 92 | 9 | Winter | 0.289865 | multidrug | 50 | 54.4 |
| GZ24 | 291 | 27 | Winter | 0.425974 | multidrug | 56 | 19.2 |
| GZ25 | 164 | 21 | Winter | 0.220538 | multidrug | 61 | 37.2 |
| GZ3 | 213 | 17 | Summer | 0.402837 | multidrug | 45 | 21.1 |
| GZ4 | 172 | 19 | Summer | 0.371297 | multidrug | 26 | 15.1 |
| GZ5 | 335 | 24 | Summer | 0.100932 | multidrug | 87 | 26.0 |
| GZ6 | 229 | 24 | Summer | 0.214351 | multidrug | 51 | 22.3 |
| GZ7 | 156 | 17 | Summer | 0.267096 | multidrug | 51 | 32.7 |
| GZ8 | 158 | 20 | Summer | 0.0535 | multidrug | 47 | 29.8 |
| GZ9 | 144 | 19 | Summer | 0.452901 | multidrug | 20 | 13.9 |
| GZ10 | 86 | 17 | Summer | 0.112939 | aminoglycoside | 8 | 9.30 |
| GZ12 | 174 | 18 | Winter | 0.021484 | aminoglycoside | 5 | 2.87 |
| GZ24 | 291 | 27 | Winter | 0.274565 | aminoglycoside | 4 | 1.37 |
| GZ25 | 164 | 21 | Winter | 0.140372 | aminoglycoside | 4 | 2.44 |
| GZ3 | 213 | 17 | Summer | 0.093932 | aminoglycoside | 6 | 2.82 |
| GZ4 | 172 | 19 | Summer | 0.126631 | aminoglycoside | 4 | 2.33 |
| GZ5 | 335 | 24 | Summer | 0.026939 | aminoglycoside | 3 | 0.89 |
| GZ7 | 156 | 17 | Summer | 0.101429 | aminoglycoside | 5 | 3.21 |
| GZ8 | 158 | 20 | Summer | 0.094538 | aminoglycoside | 12 | 7.59 |
| GZ8 | 158 | 20 | Summer | 0.007232 | glycopeptide | 1 | 0.63 |

The number of patients, AMR health-care associated infections (HAIs), and the abundance of PM_2.5_-ARGs on the sampling day are listed in **Table S3**. The portion values refer to the percentage of each type of HAIs among all detected AMR infection cases on the sampling day. Notably, beta-lactam resistance and multidrug resistance were the most abundant cases of AMR infection in the selected hospital inpatient department. These two types of HAIs were determined to account for 50.4 ± 23.6% and 44.8 ± 9.73% of all reported cases, respectively. By contrast, vancomycin (glycopeptide; 0.6%; n=1 case) and aminoglycoside (3.65 ± 2.58%, n=39 cases) AMR infection cases were two minor HAIs reported during the sampling period (**Table S3**).

For each type of resistance on the sampling day, the number of cases of infection was normalized to the number of infected patients to represent the incidence rate of antibiotic resistant HAIs. At the level of ARGs, we observed no significant correlations between the abundance of total ARGs and incidents of all AMR infections (*P* > 0.05; **Fig. S7a**). In addition, the infection cases were summarized on a weekly basis. As such, the HAI incidence rates across three days before and after the PM_2.5_ sampling day were averaged and the mean values were used in comparisons with the abundance of airborne PM_2.5_ ARGs (**Fig. S7b**). However, we still detected no significant correlations either during the whole sampling period (Pearson, *P* = 0.15) or in separate seasons (*P* > 0.05).


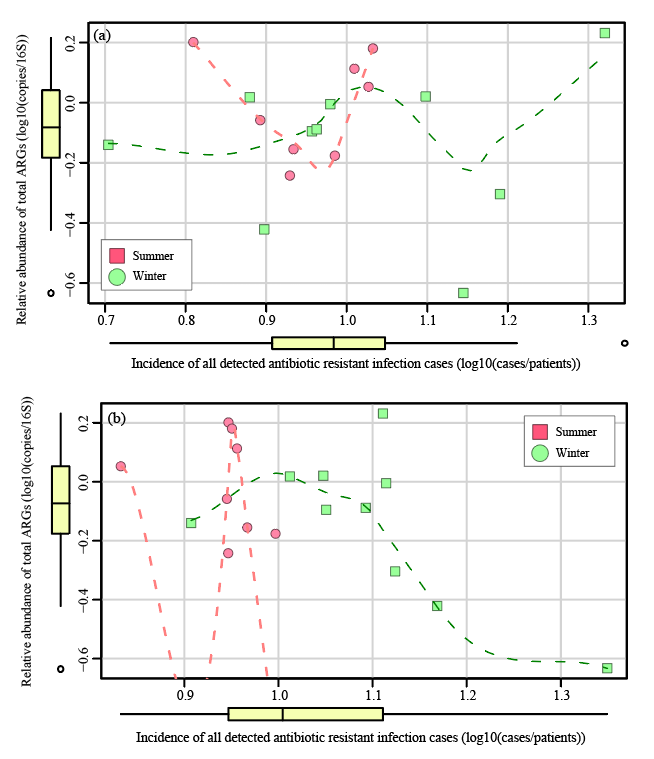


**Fig. S7** Correlations between the identified AMR infection incident rate and the relative abundance of total PM_2.5_-ARGs hosted in hospital air PM_2.5_ samples. The cases of resistant HAIs were analyzed on a daily (**a**) and weekly basis (**b**). The dashed curves generally describe the trend in the variance of the datasets by locally estimated scatterplot smoothing (span=0.75).

**Fig. S7** shows the rates of incidence of specific AMR infections (HAIs) that belonged to four resistant types (**Table S3**). In the beta-lactam resistance group, HAIs included carbapenem-resistant *Enterobacteriaceae* (CRE), extended-spectrum beta-lactamases (ESBLs), methicillin-resistant *Staphylococcus aureus* (MRSA), methicillin-resistant coagulase-negative *Staphylococci* (MRCNS), β-LAC(+), and their combined AMR cases. In the multidrug resistance group, the in-ward HAIs included multidrug-resistant *Acinetobacter baumannii* (MDRAB), multidrug resistant *Pseudomonas aeruginosa* (MDRP), and extensive drug resistant *Acinetobacter baumannii* (XDRAB). High-level aminoglycoside resistance (HLAR) and vancomycin-resistant *Enterococcus* (VRE) were also detected with a lower occurrence. **Fig. S7** shows that beta-lactam and multidrug resistance infections were reported as the major AMR cases in the hospital during the entire sampling period. The CRE and ESBL cases were major components of the beta-lactam resistant type (> 60%). At the same time, the multidrug resistant type was primarily comprised of MDRAB and MDRP. Fewer than 10 cases of the two other types of multidrug resistance were detected, and vancomycin resistant cases were only reported in one sampling day.


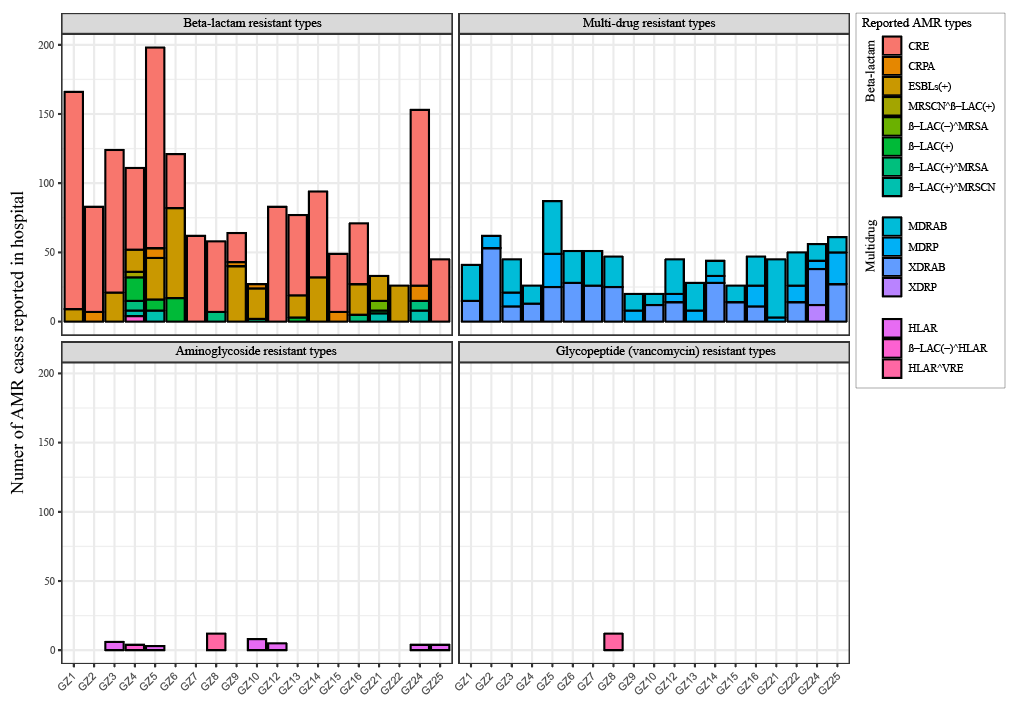


**Fig. S8** Number of detected cases of AMR infections in the hospital (inpatient department-HAIs)

To compare this data on HAIs with antibiotic use, information about which was provided monthly (**Table 1**), the daily HAIs were summarized into their sampling month (**Table S2**). The reported HAI cases were significantly higher in summertime (Mann–Whitney U-test, P = 0.05), particularly the beta-lactamase ones (362±105 vs. 219±55.1 cases/month). The aminoglycoside and glycopeptide resistant HAIs were only detected in summer (**Fig. S8**).

# SI-3 Supplementary information on statistics and sequencing data sources

In this study, various statistical analysis methods were used. Before the statistical analysis, the target datasets were initially processed in SPSS (IMB, USA) to detect the extreme value points (2.5% - 97.5%), and the outliners were removed. Descriptive statistics of all data, which included the median, mean, and standard deviation values, were performed via Excel 2010 (Microsoft Corp., USA). To enhance the distributional normality, the testing datasets were either log-transformed or scaled (mean = 0), thereby improving their fitness for a parametric and matrices correlation analysis. However, for those datasets that did not fit a normal distribution after transformation, nonparametric methods were utilized.

For all analyses/tests employed in this study, the statistical significance was always defined by 95% confidence intervals (*P* < 0.05). Specifically, regarding the datasets that fit a normal distribution, the parametric methods including Two/One-way ANONA, t-tests, and pairwise t-test were used to compare the significant difference of the mean values across different groups of data. With respect to the non-normal distribution datasets, Wilcoxon Signed Rank and Kruskal Wallis test were used. Statistical significance was always defined by 95% confidence intervals (P < 0.05). The linear correlation analysis between two groups of data was conducted using Pearson and Spearman methods concerning the parametric and non-parametric datasets. Data clustering analyses were conducted to determine differences across variable groups. This included a principal coordinate analysis that was used with multidimensional scaling of the target matrix (*e.g.,* the structure of the bacterial community and compositions of resistomes), the dissimilarity distance transformed matrix were used to conduct the permutational multivariate analysis of variance analysis (PERMANOVA) among different groups. In addition, to analyze the correlations between two selected datasets between antibiotic resistome and bacteria community, the Procrustes analysis was employed [3, 4].

To understand the overall effect of variable groups on variations in the target datasets, such as the resistome, the variance partitioning analysis (VPA) was used to quantify the contributions of each variable. Prior to this, a forward stepwise “redundancy analysis” (RDA) was used to select and screen the qualified variables, whose correlations with the target (explained dataset) matrix were significant (*P* < 0.05). For the dominant variable group, the specific effect of each variable was further calculated using generalized boosted regression models. To further explore the direct and indirect relationships among airborne beta-lactam resistant PARB, environmental variables, and health-care associated infection (HAI) cases, structural equation modeling (SEM) was built with the “lavaan” package.

**Table S4** Variation portioning analysis (VPA) table

| ***Explanation variables** | **#Df** | **R-square** | **Adjusted R-square** | **#Testable** |
| --- | --- | --- | --- | --- |
| X1 | 4 | 0.794 | 0.753 | TRUE |
| X2 | 3 | 0.410 | 0.209 | TRUE |
| X3 | 6 | 0.509 | 0.297 | TRUE |
| X1+X2 | 7 | 0.886 | 0.828 (without X3) | TRUE |
| X1+X3 | 10 | 0.930 | 0.862 (without X2) | TRUE |
| X2+X3 | 9 | 0.644 | 0.264 (without X1) | TRUE |
| X1+X2+X3 | 13 | 0.959 | **0.889** | TRUE |
| **Individual fractions** |  |  |  |  |
| X1\| X2+X3 | 4 | N/A | **0.525** (only X1) | TRUE |
| X2\| X1+X3 | 3 | N/A | **0.027** (only X2) | TRUE |
| X3\| X1+X2 | 6 | N/A | **0.061** (only X3) | TRUE |

# Fractions with the “TRUE” testable indicator was listed; the degree of freedom is denoted as Df.

*Variables were tested with no co-linearity between each other in the same group, and no redundancy was observed.

Here, we grouped these clinical and environmental datasets as potential variables to analyze their contributions to the variations in the beta-lactam resistance type of potential antibiotic resistant bacteria (PARB) by using the VPA. Together with data on beta-lactam AMR infections (**Table S3**), ambient air conditions explained only about 20% of the pattern of occurrence of airborne beta-lactam genomic ARGs (VPA, **Table S4**). This could suggest that the relationship between hospital AMR cases and PM_2.5_-ARGs could be also influenced by atmospheric environmental conditions [5].

In specific cases, as shown in **Table S4**, adjusted R-square values were used. Here, X1, X2, and X3 represent infection cases (beta-lactam, **Fig. S6**), weather, and air quality parameters (**Table S2**), respectively. About 88% of the variations in beta-lactam resistant ARGs (both genomic and total airborne) could be explained by the selected three groups, among which the infection cases as an explanatory group were found to impose the highest effects (52.5%, **Table S4**). By contrast, the direct effects of air/environmental conditions were considered minor, both of which were only ~ 5% (0.02 – 0.06). Together with AMR infections, the co-effects of environmental factors were still only estimated at 0.209 (20.9%, **Table S4**).

As such, the AMR (beta-lactam) infection cases were selected as the determinant group for further analysis, where the generalized boosted regression (gbr) method was used. As shown in **Fig. S9**, the relative influence of CRE cases was the greatest among all variables in this key (HAIs) group. This suggests that carbapenem resistance could be the most influential type of AMR infection to the airborne antibiotic resistome in hospitals. As shown in **Table S5**, the assembled metagenomes, classified as the hosting bacteria of beta-lactam resistance genes, belonged to the phyla of *Proteobacteria* and *Firmicutes*. Notably, although the winter samples exhibited no significant correlations between β-lactams resistant HAIs and related ARGs/PARB (**Fig. 5a**), bacterial taxa including *Enterobacteriaceae Providencia* and *Staphylococcaceae Staphylococcus* were still detected to carry carrying ARGs including *bla*_OXA_, penA, and *bla*z, encoding extended-spectrum β-lactamases (**Table S5**). However,


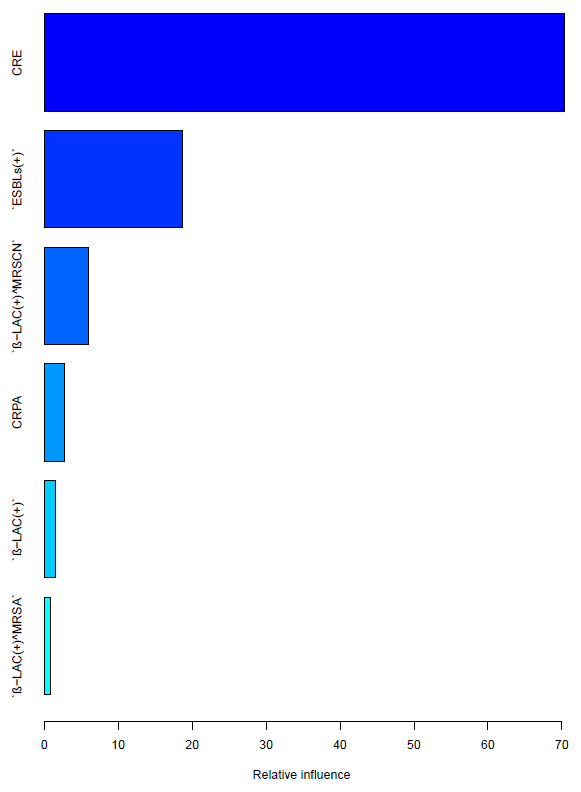


**Fig. S9** Relative influence of variables in the groups of (beta-lactam) AMR infections

**Table S5** Taxonomic classification of the assembled potential beta-lactam resistant metagenomes

| Season | Avg_fold | Classification | ARGs |
| --- | --- | --- | --- |
| Summer | 1.3966 | p__Proteobacteria;c__Gammaproteobacteria;o__Enterobacterales;f__Enterobacteriaceae;g__Pantoea;s__Pantoea dispersa | ampH |
|  | 0.1597 | p__Proteobacteria;c__Gammaproteobacteria;o__Enterobacterales;f__Enterobacteriaceae;g__Pantoea;s__Pantoea dispersa | penA |
|  | 0.37 | p__Proteobacteria;c__Gammaproteobacteria;o__Enterobacterales;f__Enterobacteriaceae;g__Pantoea;s__Pantoea dispersa | pbp2 |
|  | 5.4271 | p__Proteobacteria;c__Gammaproteobacteria;o__Pseudomonadales;f__Moraxellaceae;g__Acinetobacter;s__Acinetobacter junii | Mbl |
|  | 7.6788 | p__Proteobacteria;c__Gammaproteobacteria;o__Xanthomonadales;f__Xanthomonadaceae;g__Pseudoxanthomonas_A;s__ | AIM-1 |
|  | 17.9055 | p__Proteobacteria;c__Gammaproteobacteria;o__Burkholderiales;f__Burkholderiaceae;g__Ralstonia;s__ | OXA-22 |
|  | 21.6822 | p__Proteobacteria;c__Gammaproteobacteria;o__Burkholderiales;f__Burkholderiaceae;g__Ralstonia;s__Ralstonia insidiosa | OXA-22 |
|  | 19.6876 | p__Proteobacteria;c__Gammaproteobacteria;o__Burkholderiales;f__Burkholderiaceae;g__Ralstonia;s__Ralstonia insidiosa | OXA-60 |
|  | 26.7725 | p__Bacteroidota;c__Bacteroidia;o__Flavobacteriales;f__Weeksellaceae;g__Soonwooa;s__ | OXA-347 |
| Winter | 76.6651 | p__Proteobacteria;c__Gammaproteobacteria;o__Enterobacterales;f__Enterobacteriaceae;g__Providencia;s__Providencia stuartii_A | penA |
|  | 9.1176 | p__Proteobacteria;c__Alphaproteobacteria;o__Rhizobiales;f__Beijerinckiaceae;g__Methylobacterium;s__Methylobacterium aquaticum | ampS |
|  | 2.1894 | p__Proteobacteria;c__Gammaproteobacteria;o__Pseudomonadales;f__Moraxellaceae;g__Acinetobacter;s__Acinetobacter junii | Mbl |
|  | 29.779 | p__Proteobacteria;c__Gammaproteobacteria;o__Burkholderiales;f__Burkholderiaceae;g__Ralstonia;s__Ralstonia insidiosa | OXA-22 |
|  | 26.0824 | p__Proteobacteria;c__Gammaproteobacteria;o__Burkholderiales;f__Burkholderiaceae;g__Ralstonia;s__Ralstonia insidiosa | OXA-60 |
|  | 11.4376 | p__Proteobacteria;c__Gammaproteobacteria;o__Burkholderiales;f__Burkholderiaceae;g__Ralstonia;s__ | OXA-60 |
|  | 4.0568 | p__Firmicutes;c__Bacilli;o__Bacillales;f__Bacillaceae_H;g__Bacillus_C;s__Bacillus_C megaterium_A | BcI |
|  | 13.7837 | p__Firmicutes;c__Bacilli;o__Staphylococcales;f__Staphylococcaceae;g__Staphylococcus;s__Staphylococcus arlettae | PC1 penam |


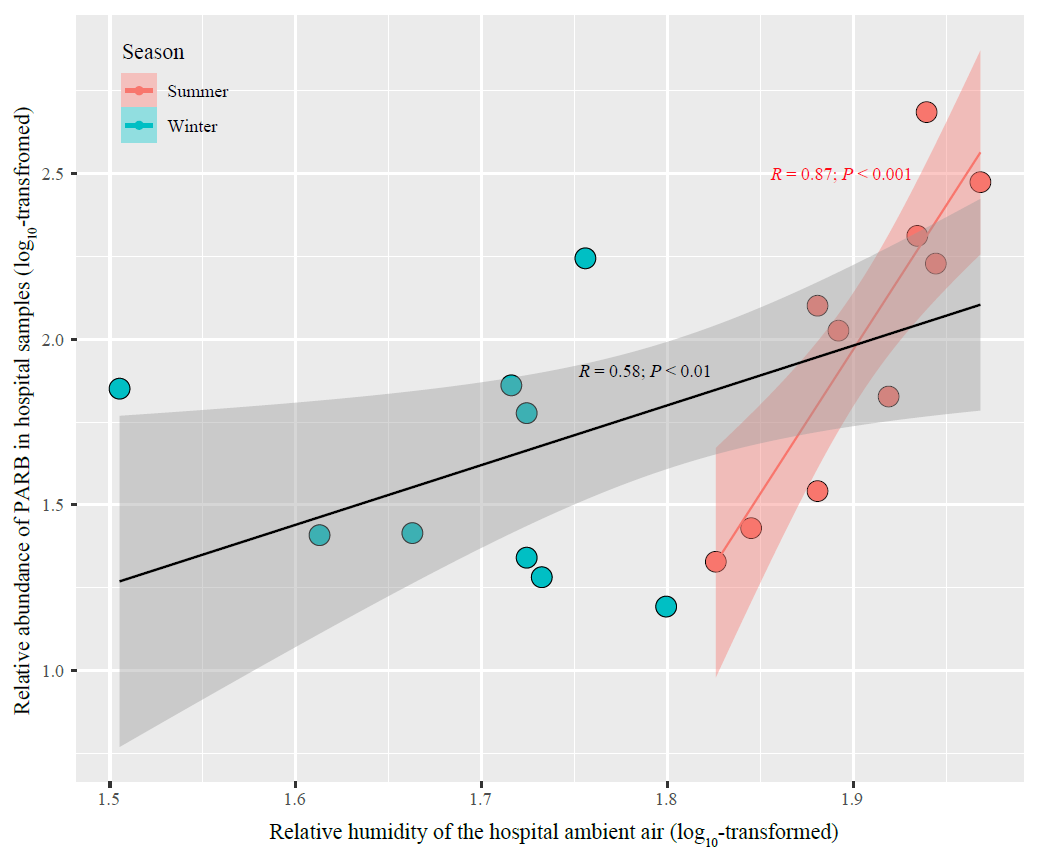


**Fig. S10** Correlations between identified PARB in hospital-specific PM_2.5_ and the relative humidity in the ambient metagenomes

As shown in **Fig. 5c**, the structure equation model suggested that the relative humidity had the highest relationship coefficient as 1.05, the highest in the group of environmental factors. This is consistent with the observed linear relationship between RH values on the sampling day and the corresponding concentrations of PARB in the hospital in the sampled PM_2.5_ (**Fig. S10**). Specifically, the relative humidity (RH) was significantly correlated to the relative abundance of hospital-emitted PM_2.5_ borne PARB (P < 0.01, **Fig. S10**), especially during the summertime (Pearson, R = 0.87, *P* < 0.001)

**Table S6** Specific information on metagenomic sequencing data retrieved from the literature

| **Sample/Project**  **Accession number** | | **Clean_Reads**  **Size (Gb)** | | **Environment_Type** | | **Data_Base** | |
| --- | --- | --- | --- | --- | --- | --- | --- |
| PRJNA436039 | | 134 | | Urban airborne particulates | | NCBI; environment  https://www.ncbi.nlm.nih.gov/ | |
| PRJNA625582 | | 90.8 | | Hospital airborne particulates winter | | NCBI; environment  https://www.ncbi.nlm.nih.gov/ | |
| SRR6797136  SRR6797141  SRR6797149  SRR6797150 SRR6797151  SRR6986811  SRR6986814  SRR6986810 | PRJNA305188 | 71.8 | Drinking/tap water | | NCBI; environment  https://www.ncbi.nlm.nih.gov/ | |  |

This study clearly shows airborne ARGs and PARB being efficiently carried by fine particulates from hospital ventilation systems, resembling in-ward HAI cases (**Fig 5a&b**). However, there was a limited set of metagenomic data for conducting a comparative investigation of airborne resistomes across different air environments in urban areas [6]. To conduct a comparative analysis to hospital-specific samples in the same geographic location, urban ambient air PM_2.5_ samples collected from Guangzhou City including Tianhe (TH) and Chonghua (CH) Districts from April of 2016 to May of 2017, which were preserved in the lab at – 40 ℃, were used in this study (**Table S6**). Apart from air-PM_2.5_ samples, metagenomic sequencing data of drinking water were selected from a global-scale study [7] were retrieved and used to compare the abundance of antibiotic resistomes with hospital PM_2.5_. Only the data from Hong Kong, Singapore, Shanghai, Macau, California, and Johannesburg were used in our analysis to represent, as much as possible, the global average profile of antibiotic resistomes in drinking water.

**Eq. S1** shows the quantification equations used for the quantification of target genes, which were initially predicted as the ARGs-like ORFs by the following protocol: using Prodigal (v2.6.3; -c -p meta mode) to predict open reading frames (ORFs) [8]. With the application of CD – HIT (v4.6) [9], these ORF sequences were further clustered under the criteria of 90% identity over the ORFs with > 90% coverage in the length of the sequences (> 250 bp). The clustered ORFs were aligned with existing ARGs (v1.1.1.A.fasta; <https://bench.cs.vt.edu/ftp/argminer/release/>) and MGE databases (https://bench.cs.vt.edu/ftp/data/databases/) via DIAMOND (v2.0.9) with parameters of alignment = 1, threshold value = 10-e7, identity > 70%, and query coverage > 50%.

Abundance ( coverage , ×/Gb) $=\frac{Map.numer \times Read.number}{Targe-gene. length\times Size}$ (**Eq. S1**)

| **Target sites** | **Harboring ARGs** | **Mean ± SD (genome/volume)** |
| --- | --- | --- |
| Hospital | Total | 2.89 ± 3.65 |
|  | MGE-associated | 1.12 ± 1.81 |
| Urban | Total | 0.49 ± 0.54 |
|  | MGE-associated | 0.18 ± 0.12 |
| Drinking water | Total | 1.42 ± 0.28 |
|  | MGE-associated | 1.00 ± 0.17 |

**Table S7** Mean concentrations of the virulent potential antibiotic resistant bacteria (HVF - PARB) genomes in airborne PM_2.5_ and drinking water

#MetaCompare pipelines were used to calculate the relative AMR risk index values [10].

As shown in **Table S7**, the mean and standard deviations of virulent potential antibiotic resistant bacteria (HVF-PARB) in different sampling sites were calculated (**Eq. S2**). These detected HVF-PARB were further classified into two categories (MGE-associated and none-MGE-associated) based on the types of ARGs that they hosted.

$HVF-PARB.site=\sum_{i=1}^{n} \frac{{{mapping.reads}_{sample-i}\times abundance}_{site}}{sample.volume \times1,000,000}/n$ (**Eq. S2**)

In **Eq. S2**, the mapping.reads indicated the number of reads used for the metagenomic binning in each sample. The abundance.site represented the averaged relative abundance of detected HVF-PARB (genome-copies/ppm-reads) in each sampling site, which was further multiplied with the mapping-read numbers of each sample in the same sampling site. For the PM_2.5_ and drinking water samples, the units of HVF-PARB concentration are denoted as genome-copies/m^3^-air and genome-copies/L-water, respectively. To normalize the sequencing depth, the resultant concentration from Eq. S2 were divided by the size of sequencing file (Gb).

**Table S7** shows that the hospital had the highest concentration (1.46 ± 0.20 genome-copies/m^3^-air) of HVF-PARB that host mobile ARGs (MGE-associated) among all source-influenced PM_2.5_ sites (One-way ANOVA, F = 30.8, *P* < 0.001). The value was twice that of PM_2.5_ in urban ambient air. Notably, the selected drinking water samples also contained the target HFV-PARB, the concentration of which was 1.00 genome-copies/L. The ingestion of drinking water is a major environment-human interaction pathway from the perspective of ‘One-Health’ [11], and the intake rate of HVF-PARB was calculated according to **Eq. S3**.

$Intake-rate.site=intake.volume \times HVF-PARB.site$ (**Eq. S3**)

Here, the intake volume of drinking water was defined as 2 liters per adult per day (not including the water intake from food [12]), and the inhalation of air was estimated as 15 m^3^ per adult per day.

# References

1. Jiang W, Liang P, Wang B, Fang J, Lang J, Tian G, Jiang J, Zhu TF: **Optimized DNA extraction and metagenomic sequencing of airborne microbial communities.** *Nat Protoc* 2015, **10:**768-779.

2. Arango-Argoty G, Garner E, Pruden A, Heath LS, Vikesland P, Zhang L: **DeepARG: a deep learning approach for predicting antibiotic resistance genes from metagenomic data.** *Microbiome* 2018, **6:**23.

3. Forsberg KJ, Patel S, Gibson MK, Lauber CL, Knight R, Fierer N, Dantas G: **Bacterial phylogeny structures soil resistomes across habitats.** *Nature* 2014, **509:**612-616.

4. Su JQ, Wei B, Ou-Yang WY, Huang FY, Zhao Y, Xu HJ, Zhu YG: **Antibiotic resistome and its association with bacterial communities during sewage sludge composting.** *Environ Sci Technol* 2015, **49:**7356-7363.

5. MacFadden DR, McGough SF, Fisman D, Santillana M, Brownstein JS: **Antibiotic resistance increases with local temperature.** *Nat Clim Chang* 2018, **8:**510-514.

6. Pal C, Bengtsson-Palme J, Kristiansson E, Larsson DG: **The structure and diversity of human, animal and environmental resistomes.** *Microbiome* 2016, **4:**54.

7. Ma L, Li B, Jiang XT, Wang YL, Xia Y, Li AD, Zhang T: **Catalogue of antibiotic resistome and host-tracking in drinking water deciphered by a large scale survey.** *Microbiome* 2017, **5:**154.

8. Hyatt D, Chen GL, Locascio PF, Land ML, Larimer FW, Hauser LJ: **Prodigal: prokaryotic gene recognition and translation initiation site identification.** *BMC Bioinformatics* 2010, **11:**119.

9. Li W, Godzik A: **Cd-hit: a fast program for clustering and comparing large sets of protein or nucleotide sequences.** *Bioinformatics* 2006, **22:**1658-1659.

10. Oh M, Pruden A, Chen C, Heath LS, Xia K, Zhang L: **MetaCompare: a computational pipeline for prioritizing environmental resistome risk.** *FEMS Microbiol Ecol* 2018, **94**.

11. Vikesland PJ, Pruden A, Alvarez PJJ, Aga D, Burgmann H, Li XD, Manaia CM, Nambi I, Wigginton K, Zhang T, Zhu YG: **Toward a comprehensive strategy to mitigate dissemination of environmental sources of antibiotic resistance.** *Environ Sci Technol* 2017, **51:**13061-13069.

12. Institute of Medicine: **Dietary Reference Intakes for Water, Potassium, Sodium, Chloride, and Sulfate. Institute of Medicine Panel on Dietary Reference Intakes for Electrolytes and Water, Standing Committee on the Scientific Evaluation of Dietary Reference intakes.** Washington D.C.: National Academy of Sciences; 2005.
